# Parenting Stress and Resilience in Parents of Children With Autism Spectrum Disorder (ASD) in Southeast Asia: A Systematic Review

**DOI:** 10.3389/fpsyg.2018.00280

**Published:** 2018-04-09

**Authors:** Kartini Ilias, Kim Cornish, Auretta S. Kummar, Miriam Sang-Ah Park, Karen J. Golden

**Affiliations:** ^1^Department of Psychology, Jeffrey Cheah School of Medicine and Health Sciences, Monash University Malaysia, Bandar Sunway, Malaysia; ^2^Department of Basic Sciences, Faculty of Health Sciences, Universiti Teknologi MARA, Puncak Alam Campus Selangor, Shah Alam, Malaysia; ^3^Faculty of Medicine, Nursing, and Health Sciences, School of Psychological Sciences, Monash University, Melbourne, VIC, Australia; ^4^School of Social & Health Sciences, Leeds Trinity University, Leeds, United Kingdom

**Keywords:** parenting stress, coping, ASD, Autism spectrum disorder, South East Asia, culture, parent, social support

## Abstract

**Background:** This paper aimed to review the literature on the factors associated with parenting stress and resilience among parents of children with autism spectrum disorder (ASD) in the South East Asia (SEA) region.

**Methods:** An extensive search of articles in multiple online databases (PsycNET, ProQuest, PudMed, EMBASE, CINAHL, Web of Science, and Google Scholar) resulted in 28 papers that met the inclusion criteria (i.e., conducted in the SEA region, specific to ASD only, published in a peer-reviewed journal, full text in English). Studies found were conducted in the following countries: Brunei, *n* = 1; Indonesia, *n* = 2; Malaysia, *n* = 12; Philippines, *n* = 5; Singapore, *n* = 5, Thailand, *n* = 2; and Vietnam, *n* = 1, but none from Cambodia, East Timor, Laos, and Myanmar were identified.

**Results:** Across the studies, six main factors were found to be associated with parenting stress: social support, severity of autism symptoms, financial difficulty, parents' perception and understanding toward ASD, parents' anxiety and worries about their child's future, and religious beliefs. These six factors could also be categorized as either a source of parenting stress or a coping strategy/resilience mechanism that may attenuate parenting stress.

**Conclusion:** The findings suggest that greater support services in Western countries may underlie the cultural differences observed in the SEA region. Limitations in the current review were identified. The limited number of studies yielded from the search suggests a need for expanded research on ASD and parenting stress, coping, and resilience in the SEA region especially in Cambodia, East Timor, Laos, and Myanmar. The identified stress and resilience factors may serve as sociocultural markers for clinicians, psychologists, and other professionals to consider when supporting parents of children with ASD.

## Introduction

Parenting children with autism spectrum disorder (ASD) can be more stressful and challenging than parenting children with typical development, especially in countries where there is a dearth of various support resources. Across the literature, parents of children with ASD frequently reported higher levels of anxiety (e.g., Stein et al., 2011; Kuusikko-Gauffin et al., 2013; Falk et al., 2014), higher levels of depression (e.g., Stein et al., 2011; Hayes and Watson, 2013; Zablotsky et al., 2013; Falk et al., 2014; Weitlauf et al., 2014), and more health-related problems (e.g., Stein et al., 2011; Dykens and Lambert, 2013; Giallo et al., 2013; Fairthorne et al., 2015). Group comparison research further found parents of children with ASD to have higher levels of stress and lower level of well-being than parents of typically developing children (e.g., Dabrowska and Pisula, 2010; Estes et al., 2013; Hayes and Watson, 2013) and/or parents of children with other developmental disabilities, such as Down syndrome (e.g., Dabrowska and Pisula, 2010; Wang et al., 2011; Dykens and Lambert, 2013; Estes et al., 2013).

Commonly, the sources of stress in parents of children with ASD include the child's inappropriate and unpredictable behaviors/emotional problems (e.g., Tomanik et al., 2004; Herring et al., 2006; Lecavalier et al., 2006; Osborne and Reed, 2009; Estes et al., 2013), severity of the autism symptoms (e.g., Osborne and Reed, 2009; Ingersoll and Hambrick, 2011; Rivard et al., 2014), as well as financial worries secondary to the need to spend for treatment intervention and education (e.g., Sharpe and Baker, 2011; Vohra et al., 2014; Zablotsky et al., 2014; Thomas et al., 2016). Studies have additionally documented the critical role that social support plays in aiding parents of children with ASD to successfully cope with their higher levels of stress (e.g., Tehee et al., 2009; Ekas et al., 2010; Lovell et al., 2012; Weiss et al., 2013); including the importance to gain easy access to and support from mental health professionals (e.g., Mackintosh et al., 2012; Vohra et al., 2014).

When distinguishing between the relative importance of factors that may contribute to parenting stress, research found that the child's emotional and behavioral problems contributed significantly more to mothers' stress, perceived family dysfunction, and parent mental health problems, than the child's dignosis, presence of a development delay, or child's gender (Herring et al., 2006). These findings highlights the importance of examining the underlying sources and predictors that may lead to parenting stress and warn us against simply assuming that the mere diagnosis of ASD in a child is sufficient as a driving factor in increasing parenting stress (Herring et al., 2006). Similarly, findings from another study suggested that regardless of child's age or gender or autism symptom severity, behavioral problems (i.e., higher levels of child hyperactivity) predicted higher parenting levels of distress (McStay et al., 2014).

There have also been mixed findings in regards to the differences in stress levels experienced by mothers and fathers. Whilst some studies have found increased stress levels to be experienced by mothers and fathers as a couple (e.g., Dabrowska and Pisula, 2010; Ingersoll and Hambrick, 2011; Harper et al., 2013), others have instead determined stress levels among mothers and fathers as separate individuals and have found parental differences in the levels of stress experienced. For example, a study found mothers' stress levels to be more greatly affected (Herring et al., 2006), whereas the findings from another study found fathers to report higher levels of stress (Rivard et al., 2014).

When examining families who have a child with ASD, cultural factors are also very important to consider (e.g., Daley et al., 2013; Freeth et al., 2014). In many cases, there is a lack of information about ASD in the society, which may lead to parents of children with ASD to face stigmas, and be influenced by cultural beliefs and/or experience self-blame for their child's diagnosis (e.g., Gray, 2002; Mak and Kwok, 2010; Neely-Barnes et al., 2011; Ravindran and Myers, 2012; Sarrett, 2015; Riany et al., 2016). With the lack of support and adequte knowledge available at the societal level, these families may also struggle to make sense of their child's behavior, which may in turn increase the level of stress experienced and delay diagnosis and treatment planning for the family (Karst and Van Hecke, 2012).

The majority of studies that have investigated parenting stress in parents of children with ASD are conducted in Western or European contexts (e.g., Hayes and Watson, 2013; Ooi et al., 2016). The relatively broader research in the Western and/or European context denotes the greater awareness and support for the autism community in those countries. In contrast, the awareness of mental health and specifically ASD is still growing in the Asian region, and therefore, is still in the early stages of receiving the necessary support (Ilias et al., 2008, 2016; Sun and Allison, 2010; Neik et al., 2014). Hence, it is imperative to determine how support may be catered toward the autism community in the under-investigated Asian countries.

To achieve this, the current paper aimed to review peer-reviewed published studies conducted in Asia (limited to the South East Asian [SEA] region) in regards to parenting stress among parents with children with ASD, and therefore provide a discussion on the sources of stress among this sample, and the variables/ factors that may attentuate the levels of stress experienced by them. It is believed that findings from this review paper will be able to provide insights on the factors related to parenting stress, specific to the SEA region, an under-researched area. Furthermore, it is hoped that the findings will be able to provide a culture-specific understanding of the stress experienced by parents of chidren with ASD in the SEA region and that, the identified stress and resilience factors may serve as sociocultural markers for clinicians, psychologists, and other professionals to consider when supporting and treating parents and families of children with ASD.

## Methods

### Selection of the studies

**Figure 1:**
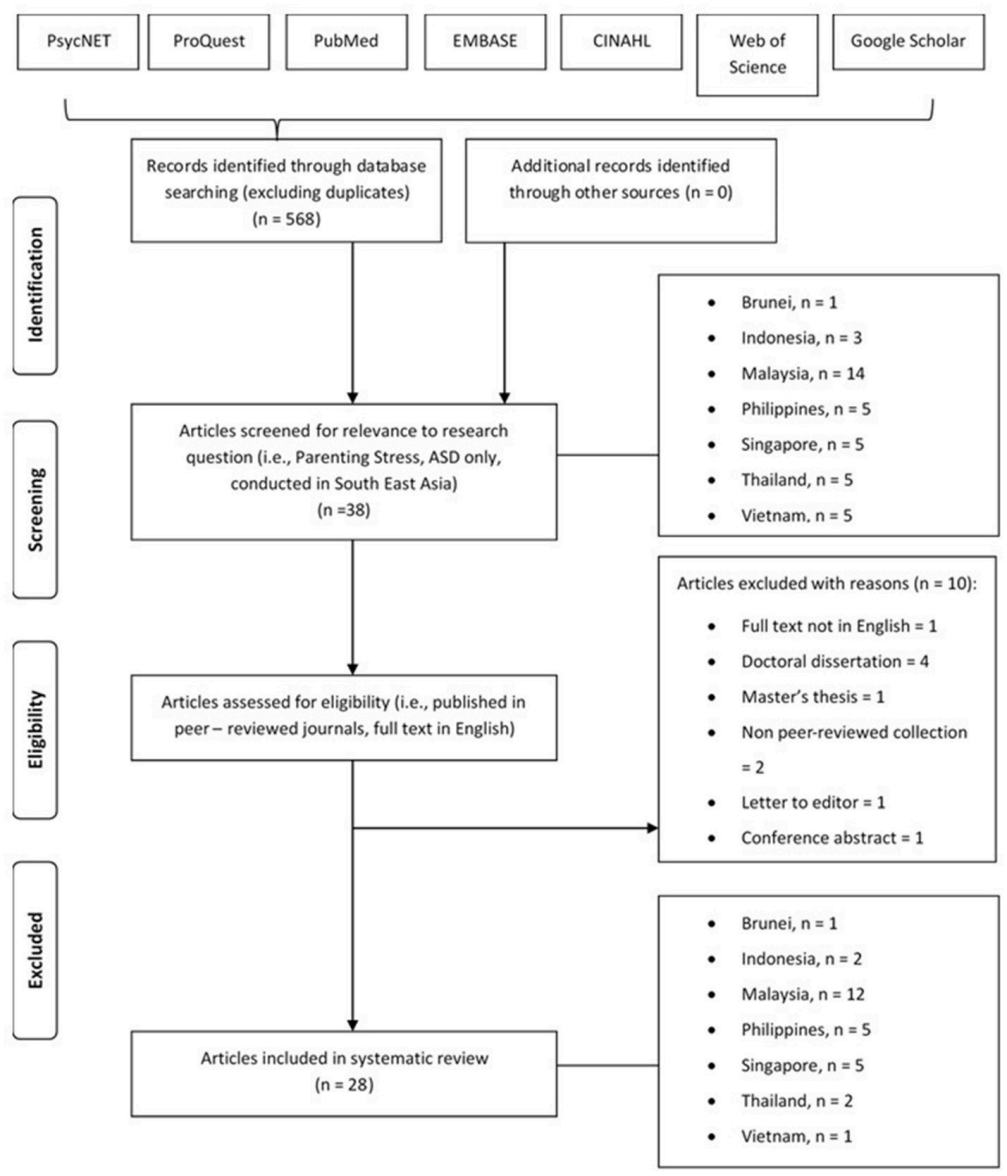
PRISMA flowchart illustrating article selection process.

The systematic review conducted on parenting stress and resilience in parents of children with ASD began with a search of literature using multiple electronic databases (PsycNET, ProQuest, PubMed, EMBASE, CINAHL, Web of Science, and Google Scholar; see Figure 1). Figure 1 was adapted from the PRISMA flow diagram (Moher et al., 2009) and the PRISMA guidelines were followed. Study authors and an external auditor, a researcher with a high level of experience with systematic reviews, reviewed the process to check the adherence with PRISMA guidelines. With an emphasis on studies conducted in SEA countries, search terms used in this initial search entailed the names of SEA countries together with the search term, “ASD” or its derivatives (e.g., autism, autistic, Asperger, pervasive development disorder). In other words, several search terms on the key concept of ASD were conducted repeatedly for each SEA country. Taking into account that some countries are referred to with other names, a second search was conducted for countries with their alternative names (e.g., Indon for Indonesia, Burma for Myanmar, Filipino for Philippines). A search for the key concept of ASD was also conducted with the search term, “South East Asia” to locate studies that might have been missed out. As a cautionary step, a final search on the key concept of parenting stress (using search terms such as “parent,” “mother,” “father,” “stress,” “resilience,” “coping”) was conducted for each SEA country to ensure no relevant studies were overlooked. Together, a total of 568 studies on ASD in respect to the SEA region were identified.

The first screening process was then conducted to exclude ASD studies in SEA countries that were unrelated to parenting stress and resilience. The chronological order of this particular step was intentional to demonstrate the comparatively smaller ratio of ASD studies in SEA countries that are specifically targeted toward studying parenting stress and resilience. Studies that additionally sampled parent(s) of children with other forms of cognitive delay(s) and/or disability(ies) other than ASD (i.e., ADHD, dyslexia, etc.) were also excluded (e.g., Shin et al., 2006; Shin and Nhan, 2009). Additionally, studies that sampled South East Asian parents, but were not conducted in South East Asian countries were also excluded (e.g., Luong et al., 2009). This ensured that the studies reviewed in this paper were specific to the cultural context of SEA countries. Therefore, this refined search led to a new total of 38 articles.

At the second and last screening process, those that did not meet the other inclusion criteria (i.e., not published in peer-reviewed journals; full text not in English; doctoral dissertation; Master's thesis) were excluded. Finally, a total of 28 studies were deemed eligible to be reviewed.

A conscious decision was made not to limit the range of years of published studies, in order not to further reduce an already narrow yield of results in this under-researched region. However, it was planned to exclude studies published before 1980, in accordance with the initial inclusion of autism in the Diagnostic and Statistical Manual, 3rd edition (American Psychiatric Association, 1980). Only one study was identified as published in the 1980s or 1990s (Liwag, 1989) and this study was retained; themes were consistent with findings in later writings. Articles published after July 2016 were not included in this review. An intentional decision was made to focus on peer-reviewed articles and the search was not inclusive for theses/dissertations. It was judged that including theses/dissertations would introduce bias through the identification of some theses/dissertations over others, depending on their language of writing and online availability.

Before the search commenced, study authors agreed on the search strategy in consultation with a senior librarian advisor. A senior librarian advisor was also consulted throughout the search process. Moreover, an external auditor reviewed the process involved in the selection of studies before and after the study.

### A-priori features of interest among reviewed studies

Studies included in this review are presented in Supplementary Table 1. Features of the studies as summarized in the header of Supplementary Table 1 include: location, design/method, sample, outcome measure, aim, findings, and control variable. The descriptor, “location” was included to allow face-value comparisons of the studies amongst SEA countries. The “design/method” of the study enables a quick evaluation of the methodology in which the study was conducted (i.e., quantitative, qualitative, or mixed-method), and thus, how that would relate to its findings. The header, “sample” specifies the number of participants in the study, if a particular study used a comparison group (i.e., parents of children with typical development), and if both mothers and fathers, or only single parents were sampled. The “outcome measure” looks at the utilization of differential measures across the reviewed studies in measuring common variables such as parenting stress, coping strategies, depression and anxiety symptoms, severity of autism symptoms and so forth. The “aim” describes the purpose of a particular reviewed study, while “findings” outlines key results from that study.

### Analysis procedures

Studies yielded from the search were analyzed through a careful and strategic process. Synthesis of literature findings was conducted by reading full-text articles of included studies, identifying parenting stress factors. Each study was read multiple times by four of the study authors to prevent bias. Identified factor(s) affecting parenting stress were noted and recorded for each study. Characteristics of each study, as listed in Supplementary Tables 1, 2 and Table 1 (e.g., location, design, parents' characteristics, child characteristics) were also noted and recorded. The use of objective measures, such as questionnaires were also noted to determine how parenting stress was differentially measured across studies.

**Table 1:** Characteristics of children diagnosed with ASD of parents sampled in this article review.

**Study**	***N***	**Age of children**	**Age diagnosed**	**Diagnosis**
Athari et al., 2013	Not stated.	Age range = 6–8 years.	Not stated.	Autistic Disorder
Callos, 2012	Not stated.	Not stated.	Not stated.	ASD
Charnsil and Bathia, 2010	Not stated.	Not stated.	Not stated.	Autistic Disorder
Chong and Kua, 2016	10	Age range: 6–9 years.	*M* = 2+ years.	Autistic Disorder
Foo et al., 2014	6 (4 males, 2 females).	Age range = 10–18 years (*M* = 12 years 10 months).	Not stated.	Five diagnosed with Autistic Disorder; One diagnosed with Asperger's syndrome.
Foronda, 2000	16	Not stated.	Not stated.	ASD
Ilias et al., 2016	10	Age range = 5–23 years (*M* = 11 years).	*M* = 2 years 11 months.	Autistic Disorder
Ha et al., 2014	24	Age range = 2–10 years old (*n* = 15), above 10 (*n* = 12).	Not stated.	ASD
Lai et al., 2015	136. (73 with ASD, 63 with typical development).			
77 males (56.6%), 59 females (43.4%).	*M* = 12.35 years, *SD* = 3.67 years.	Not stated.	Autistic Disorder (*n* = 43), Asperger's syndrome (*n* = 15), PDD – NOS (*n* = 15), typical development (*n* = 63).	
Liwag, 1989	13	Age range = 4–9 years.	At the time of the study, it was 3–4 years since the child was diagnosed with autism.	Early infantile autism as according to DSM-III.
Moh and Magiati, 2012	102. Out of the 98 children who reported gender, 82 were males, and 16 were females.	Age range = 2–17 years, 3months (*M* = 7 years, 3 months; *SD* = 2 years, 9months).	Out of the 93 responses received, age range: 16–96 months (*M* = 40.7 months, *SD* = 14.4 months).	Out of the 99 children who reported diagnosis: Autistic Disorder (*n* = 25), ASD (*n* = 65), Asperger's Syndrome (*n* = 2), PDD-NOS (*n* = 7).
Nikmat et al., 2008	Not stated.	Not stated.	Not stated.	ASD
Quilendrino et al., 2015	Phase 1 (Focus group discussion): 10 children (7 males, 3 females). Phase 2 (survey): 41 children (36 males, 5 females).	Phase 1 (*M* = 75.43 months, *SD* = 33.74 months). Phase 2 (*M* = 60.73 months, *SD* = 17.45 months).	Quantitative analysis from survey (*M* = 39.39 months).	Autism
Rahman et al., 2012	2 (one daughter, one son)	Daughter: 7 years. Son: 9 years.	Not stated.	Daughter: Autistic Disorder. Son: PDD-NOS, comorbid with Attention Deficit Hyperactive Disorder (ADHD).
Rejani and Ting, 2015	Not stated.	Not stated.	Not stated.	Autism
Resurreccion, 2013	10	Age range = 6–20 years; *M* = 11.9 years	Not stated.	Autistic Disorder
Roffeei et al., 2015	Not stated.	Not stated.	Not stated.	ASD
Santoso et al., 2015	14	*M* = 7.9 years	Not stated.	ASD
Siah and Tan, 2015	Male (72%), female (21%).	7 years or below (56.63%), 7 – 14 years (19.28%), above 14 years (24.09%).	Not stated.	ASD
Siah and Tan, 2016	Not stated.	Not stated.	Not stated.	ASD
Sian and Tan, 2012	Not stated.	Not stated.	Not stated.	ASD
Tait and Mundia, 2012	30	*M* = 7.4 years	Not stated.	ASD
Ting and Chuah, 2010	12	Not stated.	2 years (*n* = 2); 4–5 years (*n* = 10).	ASD
Vetrayan et al., 2013	33	Age range = 0–6 years (39.4%), 7–12 years (48.5%), 13–18 years (12.1%).	Not stated.	“Moderately autistic” (*n* = 10), “Severely autistic” (*n* = 23).
Wahyuni, 2013	2	Case study 1 = 12 years. Case study 2 = Not stated.	Case study 1 = 2 years. Case study 2 = 3 years/	Autistic Disorder
Wisessathorn et al., 2013	333 (*n* = 255, 76.6% were males).	Age range = 2–17 years; *M* = 7.83 years; *SD* = 3.46 years.	*M* = 3.59 years; *SD* = 3.46 years.	ASD
Xue et al., 2014	65 (*n* = 60, 92.3% were males).	Age range = 3.2–11.8 years (*M* = 6.9 years, *SD* = 2.1 years). One child's age was not reported.	*M* = 35.3 months (*n* = 64).	ASD (*n* = 46; 73%), Autistic Disorder (*n* = 12; 19%), Asperger's syndrome (*n* = 3; 4.8%), PDD-NOS (*n* = 2; 3.2%). The diagnoses of two children were not reported.
Yeo and Lu, 2012	Not stated.	Not stated.	Not stated.	Autistic Disorder

*M, Mean; SD, Standard Deviation*.

Similarities and differences between studies were highlighted as well as pooling together similar reporting measures and outcomes across studies. All relevant influential factors were thematically categorized and presented in a descriptive approach. All authors agreed on the data analysis findings and factor categorization. To minimize bias and promote trustworthiness of the findings, three experienced researchers with knowledge in the topic area also reviewed the data analysis findings and agreed with our factor categorization and discussion of the results.

## Results

### Description of studies reviewed in this article

A total of 28 studies were finally chosen for review in this paper (see Supplementary Table 1). Out of these 28 studies, 14 had quantitative designs, 11 were qualitative, and three were mixed-method. Out of the 14 quantitative studies however, only one (Lai et al., 2015) used an active control group (i.e., parents of children with typical development) for mean comparison.

The resulting search found relevant studies to be investigated in only seven of the total, 12 SEA countries (Brunei, *n* = 1; Indonesia, *n* = 2; Malaysia, *n* = 12; Philippines, *n* = 5; Singapore, *n* = 5, Thailand, *n* = 2; Vietnam, *n* = 1). It would however be inaccurate to infer that SEA countries that were not yielded in this search have a lower awareness of ASD. This lack of identified articles in five SEA countries may be mainly due to the possibility that factors such as funding may have played a role in the lack of/absence of published research in those countries.

Across the studies, the total sample amounted to 1, 639 participants, whereby 1, 288 were mothers and 253 were fathers. Note that two studies (Vetrayan et al., 2013; Roffeei et al., 2015) did not report the sampled number of fathers and mothers in their study. Parents in the reviewed studies had at least one child who was diagnosed with ASD. The cumulative age range of these children across all studies was 0–20 years, though it should be noted that several studies failed to report the age of the child who was diagnosed with ASD in their study. The majority of the studies specified the diagnosis of the children under the broader term, “ASD” (Diagnostic and Statistical Manual of Mental Disorders 5th ed.; DSM-5; American Psychiatric Association, 2013) whilst others followed the categorization listed under Pervasive Developmental Disorders (PDD) in the fourth edition, text revision of the DSM (DSM-IV-TR; American Psychiatric Association, 2000). Supplementary Table 2 and Table 1 display the characteristics/demographics of parents and children, respectively.

Although “autism” more accurately refers to autistic disorder as listed under PDD in the DSM-IV-TR (American Psychiatric Association, 2000), it has been used interchangeably with ASD in several of the reviewed articles to inclusively refer to other autism-related disorders besides autistic disorder. Therefore, to ensure clarity in this paper, the term “autism” was replaced with “ASD” or “autistic disorder” when its intended meaning was obvious to the authors.

### Parenting stress and other related variables

During the search process of the reviewed articles, it was noted that parenting stress among parents of children with ASD was not always examined using the term “parenting stress” per se. Rather, other related variables were also investigated, which provided an idea for different mechanisms that interact with the manifestations of stress among parents of children with ASD (e.g., resilience, psychological distress, etc.). Several of the studies investigated parenting stress as the main variable in their study (e.g., Liwag, 1989; Foronda, 2000; Nikmat et al., 2008; Moh and Magiati, 2012; Yeo and Lu, 2012; Athari et al., 2013; Xue et al., 2014; Lai et al., 2015). Some studies (Nikmat et al., 2008; Yeo and Lu, 2012; Xue et al., 2014; Lai et al., 2015) assessed “stress” through the use of objective measures (e.g., Parenting Stress Index, Abidin, 1992; Parental Stress Scale, Berry and Jones, 1995; The Questionnaire on Resources and Stress– Friedrich Short Form, Friedrich et al., 1983); whereas, other studies determined levels of parenting stress via interviews, in line with the qualitative nature of their study (Liwag, 1989; Foronda, 2000), or by calculating an index using a scale they developed (Moh and Magiati, 2012).

One of the related variables of parenting stress that was explored was level of psychological well-being/distress among parents of children with ASD (e.g., Nikmat et al., 2008; Yeo and Lu, 2012; Lai et al., 2015; Rejani and Ting, 2015). For this, measures such as the Depression, Anxiety and Stress Scale (DASS; Lovibond and Lovibond, 1995), and the General Health Questionnaire (GHQ-28; Goldberg, 1978) were utilized. The use of these scales have, therefore, highlighted the need to understand and study “stress” among parents of children with ASD, as a component of psychological distress that is compounded with other related factors such as depression and anxiety. Accordingly, other studies (Charnsil and Bathia, 2010; Rejani and Ting, 2015) were included in this systematic review, as their findings on depressive and/or anxiety symptom levels among parents of children with ASD could highlight several factors that might also be associated with parenting stress.

Other variables that were also determined in relation to parenting stress included parental satisfaction (Moh and Magiati, 2012), resilience (Santoso et al., 2015), quality of life (Sian and Tan, 2012; Wisessathorn et al., 2013; Siah and Tan, 2015, 2016), family functioning (Tait and Mundia, 2012; Xue et al., 2014), and hopelessness (Vetrayan et al., 2013). Although these variables were not identical to the concept of parenting stress, they were included in the current review to provide a holistic understanding of the stress experienced by parents of children with ASD.

Remarkably, more than half of the total reviewed studies were found to also investigate coping strategies/mechanism among parents of children with ASD (Liwag, 1989; Foronda, 2000; Ting and Chuah, 2010; Callos, 2012; Sian and Tan, 2012; Resurreccion, 2013; Wahyuni, 2013; Wisessathorn et al., 2013; Foo et al., 2014; Xue et al., 2014; Lai et al., 2015; Quilendrino et al., 2015; Roffeei et al., 2015; Santoso et al., 2015; Siah and Tan, 2015, 2016; Chong and Kua, 2016; Ilias et al., 2016). This focus area suggests that even within the SEA region, there is awareness for the need to inclusively discuss coping strategies for parents of children with ASD as opposed to merely focusing on intervention strategies for the child diagnosed with ASD. Furthermore, the current review of the coping strategies employed amongst parents of children with ASD highlights the factors that may attenuate levels of parenting stress in this population.

### Factors/variables affecting parenting stress

As a result of the review, several factors were found to have played a prominent role in the levels of parenting stress experienced by parents of children with ASD in the SEA region. Particularly, these factors included: (i) social support, (ii) severity of autism symptoms, (iii) financial difficulty, (iv) parents' understanding and perception toward ASD, (v) parents' anxiety and worries about their child's future, and (vi) religious belief.

- **Social support**. Across the studies, social support was found to be a coping mechanism that was frequently reported in easing parenting stress (Liwag, 1989; Foronda, 2000; Ting and Chuah, 2010; Callos, 2012; Moh and Magiati, 2012; Yeo and Lu, 2012; Wahyuni, 2013; Foo et al., 2014; Ha et al., 2014; Xue et al., 2014; Roffeei et al., 2015; Santoso et al., 2015; Chong and Kua, 2016). Specifically, social support was attained from numerous sources, which included support from the immediate family, schools, families of other children with ASD, professionals, and/or extended family members (e.g., Foronda, 2000; Xue et al., 2014; Santoso et al., 2015; Chong and Kua, 2016). However, in respect to the support drawn from external family members, contradictory results were found. In particular, unlike the research by Xue et al. (2014), the research by Foronda (2000) reported that parents drew less social support from extended family members, specifically, spouses' relatives (in-laws). On the other hand, spousal relationship itself was found to be an important source of social support (Foronda, 2000; Foo et al., 2014; Santoso et al., 2015; Chong and Kua, 2016), and was found to be a cross-cultural factor that significantly predicted parenting stress and psychological distress among parents of children with ASD in Malaysia as well as in China (Yeo and Lu, 2012).
  Another noteworthy finding points to the critical role professionals were suggested to play in providing social support to these parents (Liwag, 1989; Foronda, 2000; Moh and Magiati, 2012; Rahman et al., 2012; Resurreccion, 2013; Xue et al., 2014; Santoso et al., 2015; Chong and Kua, 2016). Particularly, in Singapore, a research study found that higher parental stress was associated with a higher number of professionals consulted, and a lower perceived collaboration between parent and professionals; though, only the number of professionals consulted was found to significantly predict parenting stress (Moh and Magiati, 2012). They also found that collaboration with professionals, the perceived helpfulness of the information provided, and parental stress significantly predicted parental satisfaction (Moh and Magiati, 2012).
  Overall, these findings on social support are similar to that found by a research study in Malaysia, which identified that the two types of support that parents and caregivers most frequently engaged in were informational support (i.e., professionals) and emotional support (i.e., family, friends, etc.) (Roffeei et al., 2015). Findings from this study also demonstrated that sources of social support in today's technologically advanced society now include online communities, such as Facebook (Sian and Tan, 2012). It is also likely that parents with a higher level of intrinsic motivation to participate in supportive groups and/or programs might experience enhanced social relationships, as found in the study (Sian and Tan, 2012).
- **Severity of autism symptoms**. Besides social support, severity of autism symptoms was also found to affect parenting stress among parents of children with ASD (Liwag, 1989; Foronda, 2000; Nikmat et al., 2008; Charnsil and Bathia, 2010; Yeo and Lu, 2012; Athari et al., 2013; Wisessathorn et al., 2013; Lai et al., 2015; Ilias et al., 2016). Using a comparison group (parents of children with typical development) in a Singaporean sample, a study was able to show that parents of children with ASD (who were found to have a greater experience of difficult child behavior) had higher levels of parenting stress (Lai et al., 2015). Moreover, the perception of autism severity (specifically, mother's perception) was found to be a cross-cultural factor (in Malaysia and China) that significantly predicted parenting stress. However, another study did not find ASD symptom severity to be associated with a retrospective rating of parenting stress in relation to the diagnostic process (Moh and Magiati, 2012).
  Autism severity was found to be negatively associated with related variables of parenting stress, such as parental quality of life (Wisessathorn et al., 2013) and parental satisfaction during the diagnostic process (Moh and Magiati, 2012). Similarly, a study found that severity of autism symptoms in a Thai sample was higher among children with caregivers who were clinically depressed, although autism symptoms did not predict depressive disorders among the caregivers (Charnsil and Bathia, 2010). The authors commented that the non-significant finding may be impacted by the small sample size (27 participants) (Charnsil and Bathia, 2010).
  Uniquely, in the study by Xue et al. (2014), parents reported that although they were occasionally challenged with aggressive and self-injurious behaviors that are typically stereotyped with children with ASD, they found these behaviors to have only little interference with their lives. In fact, this group of parents reported low levels of stress, contradictory to the typical findings on parenting stress among parents of children with ASD.
  On another note, the reverse of this ASD severity–parenting stress relationship was also found to be true, whereby further analyses (Athari et al., 2013) revealed that levels of stress (and depression) among mothers of children with ASD predicted the severity of autism in their child. These findings together, therefore, suggest the complexity of this relationship.
- **Financial difficulty**. Financial difficulty was a factor that was frequently reported to influence the level of stress experienced among parents of children with ASD (Liwag, 1989; Foronda, 2000; Rahman et al., 2012; Tait and Mundia, 2012; Yeo and Lu, 2012; Athari et al., 2013; Vetrayan et al., 2013; Wahyuni, 2013; Ha et al., 2014; Quilendrino et al., 2015). Financial income (the inverse of financial difficulty) was found to negatively correlate with levels of parenting stress and depression (Athari et al., 2013) as well as levels of hopelessness (Vetrayan et al., 2013) among parents of children with ASD. Financial income was also found to negatively correlate with severity of autism symptoms, and even mediated the positive relationship between parenting stress and severity of autism symptoms (Athari et al., 2013).
  From a cross-cultural perspective examining maternal parenting stress and maternal psychological distress, Yeo and Lu (2012) found treatment costs/father's income to have some differing impacts in Malaysia and China. For example, father's income/treatment costs contributed 15% of the variance in psychological distress for Malaysian mothers compared to only 3% for Chinese mothers. Treatment cost was considered to be an important factor that contributed to the difficulties and distress especially among Malaysian mothers, as it was speculated that Malaysian families are further challenged by having to support schooling needs for multiple children, in comparison to the Chinese, single-child families. Furthermore, Yeo and Lu's (2012) findings also highlight the cross-cultural experience of stress as a component of psychological distress.
- **Parents' perception and understanding toward ASD**. Besides the aforementioned factors, parents' perception and/or understanding of having a child diagnosed with ASD was also found to play an important role in regards to the parenting stress experienced. More specifically, positive beliefs/optimism (Foronda, 2000; Callos, 2012; Wisessathorn et al., 2013; Chong and Kua, 2016), emotional acceptance and understanding (Liwag, 1989; Wahyuni, 2013; Xue et al., 2014; Chong and Kua, 2016; Ilias et al., 2016), sense of coherence (Siah and Tan, 2015, 2016), cognitive reframing (Siah and Tan, 2016), and adaptability (Wahyuni, 2013; Ilias et al., 2016) toward having a child with ASD served as coping strategies for parents of children with ASD.
  On the contrary, negative perceptions of their child's diagnosis have also been reported. For example, in the case study (Rahman et al., 2012), the father, who was diagnosed with depression and was reported to engage in substance abuse (i.e., alcoholism), perceived the autism diagnoses in his children as a result of him being “cursed” for his previous bad behavior. This is in contrast to the findings of a research study by Quilendrino et al. (2015), which found parents to disagree with cultural myths and beliefs about ASD (e.g., not a result of a curse and/or parental sin). Similarly, a study found that mothers were commonly exposed to culturally transmitted fears and concerns that they may have done something wrong in the past to cause the disorder (e.g., karma or spirit possession). Even though, the mothers did not believe these traditional lay beliefs themselves, they still described these societal perspectives as stigmatizing and impacting their well-being (Ilias et al., 2016).
- **Parents' anxiety and worries about their child's future**. Several studies (via qualitative interviews or an open-ended sentence completion task), found parents' anxiety in regards to the future of their child (who is diagnosed with ASD) to be one of their sources of stress (Liwag, 1989; Foronda, 2000; Tait and Mundia, 2012; Ha et al., 2014; Quilendrino et al., 2015; Ilias et al., 2016). The parents' anxieties included the child's schooling and future secondary and/or higher education (Tait and Mundia, 2012; Ilias et al., 2016), job and career prospects (Ilias et al., 2016), and worries over finding care for their children in their older age (Quilendrino et al., 2015). Conversely, thoughts of their child's future motivated mothers to be resilient when they were optimistic about their child's future (Santoso et al., 2015).
- **Religious belief**. Several parents additionally reported religious beliefs as a coping strategy and as a support, helping them to accept and raise a child with ASD (e.g., Ting and Chuah, 2010; Tait and Mundia, 2012; Resurreccion, 2013; Wahyuni, 2013; Santoso et al., 2015; Chong and Kua, 2016; Ilias et al., 2016). For example, parents accepted their child as a gift from God, in spite of his or her ASD diagnosis (e.g., Ting and Chuah, 2010; Tait and Mundia, 2012), found comfort through prayer (e.g., Wahyuni, 2013; Santoso et al., 2015; Ilias et al., 2016), and/or by reading holy books (e.g., Resurreccion, 2013; Ilias et al., 2016) and/or through church involvement (e.g., Ilias et al.).

## Discussion

The present article aimed to review studies conducted in the SEA region, identifying the factors that influence or are associated with parenting stress among parents of children with ASD. Across the 28 studies that were included in this systematic review, four factors associated with parenting stress, were frequently reported: social support, severity of autism symptoms, financial difficulty, and parents' perception and understanding toward ASD. Two other factors were also found to relate to parenting stress, though to a lesser extent; these included parents' anxiety and worries about their child's future, and religious belief. The finding of these six factors could also be categorized in this review as either a source of parenting stress (i.e., severity of autism symptoms, financial difficulty and parents' anxiety and worries about their child's future) or as a coping strategy/mechanism that may attenuate levels of parenting stress (i.e., social support, parents' perception and understanding toward ASD and religious belief).

To a certain extent, findings from this review are comparable to that yielded from the Western context (e.g., Herring et al., 2006; Ekas et al., 2010; Ingersoll and Hambrick, 2011; Lovell et al., 2012; Mackintosh et al., 2012; Estes et al., 2013; Weiss et al., 2013; Rivard et al., 2014; Vohra et al., 2014; Zablotsky et al., 2014; Thomas et al., 2016), particularly in regards to social support, severity of autism symptoms, and financial difficulties. However, cultural variations and/or economic differences were found to underlie the differences in how these factors were uniquely manifested and/or experienced in SEA. For instance, poorer policy and economic support for mental health and special needs in the Asian region (e.g., Foronda, 2000; Ting and Chuah, 2010; Tait and Mundia, 2012; Foo et al., 2014; Ha et al., 2014; Ilias et al., 2016) may have exacerbated the difficulties and challenges faced by parents of children with ASD in these countries in comparison to those in a Western context. Moreover, stigma and discrimination toward ASD, that is relatively more prevalent in an Asian context, might influence how parents of children with ASD are further challenged (e.g., Ting and Chuah, 2010; Rahman et al., 2012; Ha et al., 2014; Ilias et al., 2016).

Furthermore, in the region, parents with lower educational and socioeconomic statuses may be further disadvantaged, identifying later the symptoms of ASD (e.g., Moh and Magiati, 2012) and facing greater risk of stress (e.g., Athari et al., 2013), depression (e.g., Charnsil and Bathia, 2010; Athari et al., 2013), and hopelessness (Vetrayan et al., 2013). As researches suggested, parents from lower socio-educational backgrounds may be a particular group to target and support in terms of education on the early warning signs of ASD and child development in general (Moh and Magiati, 2012).

In regards to the identified factors, this systematic review was able to highlight the beneficial role of social support among parents of children with ASD, and that this social support could be drawn from various sources (e.g., spouse, immediate family, external family, other families of children with ASD, schools, professionals, online). However, it was noteworthy to find contradictory results in regards to the support received by external family members (Foronda, 2000; Yeo and Lu, 2012). This difference may be due to the quality of the already existing relationship between the parent and external family members, whereby a close relationship to a member of the family, regardless if he/she is an external or immediate part of the family, could result in greater social support from him/her. In turn, this observation highlights the equally important need for the social support received to be of high quality. For instance, the findings of the spousal relationship as an important source of social support (e.g., Foronda, 2000; Yeo and Lu, 2012) might have an opposite effect if the spousal relationship was unhealthy and/or violent.

Also noteworthy is that findings on the social support received from extended family members and/or community, seem to imply it as a cultural factor that is typical of the collective and supportive culture in an Asian context (e.g., (Foronda, 2000; Charnsil and Bathia, 2010; Ilias et al., 2016). As such, future studies are recommended to explore the impact of collectivist and individualistic values in relation to the types of social support valued.

It should also be noted that despite finding social support to be a coping strategy for parents in the majority of the reviewed studies, a study found a lack of positive association between social support and parenting stress (Nikmat et al., 2008). This finding may be attributable to the difference in the measure of social support used in their study in comparison to other studies. Whilst the Provision Social Relation (PSR; Turner et al., 1983) measure as used in a study (Nikmat et al., 2008) specifically looks at the overall perception of social support received, other quantitative studies determined the sources of social support and/or their relative importance (Moh and Magiati, 2012; Yeo and Lu, 2012; Xue et al., 2014); as did qualitative studies (Liwag, 1989; Foronda, 2000; Wahyuni, 2013; Foo et al., 2014; Santoso et al., 2015; Chong and Kua, 2016). Together, this evidence might suggest that while social support may play a role in attenuating parenting stress experienced by parents of children with ASD, this finding is specific to the sources it is drawn from, rather than acting as a general perception of feeling supported. This offered interpretation, is merely preliminary based on the findings of this systematic review and would require a thorough mixed-method study (Ilias et al., 2015) to support its accuracy.

The importance of *quality* social support was also seen from an informational perspective (Roffeei et al., 2015) and was seen to include support received by professionals. Particularly, lower perceived collaboration with a professional was associated with greater stress (Moh and Magiati, 2012), whereas contact with professional help was found to subside the suicide ideation within a family who was isolated from their own family members and community whilst caring for their children with ASD (Rahman et al., 2012). As a cross-cultural observation, it is worth noting that the importance of support from professionals and/or policy makers has also been reported in other regions (mainly in the Western/European context; Ooi et al., 2016) in either creating a positive or negative experience for parents of children with ASD. However, in the SEA region, where support services are only beginning to phase into conception and early-development, there may be a greater challenge as to how these support services are attained and experienced. As a result, this lack of quality support services, might increase the stress experienced by parents of children with ASD, which is likely to be more evident in developing countries that are limited in assessment, diagnosis, intervention services, and government provisions (e.g., Ting and Chuah, 2010; Tait and Mundia, 2012; Ha et al., 2014).

As suggested earlier, the finding of “severity of autism symptoms” as a source of parenting stress among parents of children with ASD, is one that is supported by similar findings in a Western/European context. In a South-East Asian context, where funding policies underequip schools and mental health care providers with sufficient personnel and resources for the intervention of challenging behaviors in children with ASD (Phua, 2012; Poon, 2013; Ha et al., 2014), the “severity of autism symptoms” might be a relatively more salient challenge for parents of children with ASD in this region. Across the 28 studies reviewed in this paper, just one study (Xue et al., 2014) found parents of children with ASD to experience relatively low levels of stress, and to only experience a mild interference with their family life when their child with ASD occasionally exhibited stereotyped, aggressive behavior. A study inferred this atypical result likely to be due to the milder symptoms of ASD among the children sampled (Xue et al., 2014). Specifically, the authors claimed that because the majority of the children had a diagnosis of ASD without intellectual disabilities, comorbid medical conditions and severe aggressive behaviors, parents might have been better able to cope with their child's autism symptoms and thus, experienced lower levels of stress (Xue et al., 2014).

In regards to financial difficulty, there is a tacit assumption that this factor may be more specific to the stress experienced by fathers in comparison to mothers (e.g., Pisula, 2011). Accordingly, in this review, Liwag (1989) found financial difficulty to be a source of stress that was more relevant to fathers in comparison to mothers of children with ASD. This finding may presumably reflect the paternal dominance as the breadwinner of the family in an Asian context. However, given the lapse of time since this finding (30 years) and hence, the sociocultural changes and shifting gender roles since then, it is uncertain if such gender differences might still exist. Moreover, the variability in design across the studies in this review makes it difficult to determine if there are indeed gender differences in respect to this factor. For example, most studies that revealed financial difficulty to be a contributing factor of parenting stress, sampled only mothers and thus, made it impossible to determine gender differences between fathers and mothers (Foronda, 2000; Yeo and Lu, 2012; Athari et al., 2013; Wahyuni, 2013). Similarly, other studies, which also found financial difficulty to positively associate with parenting stress, analyzed fathers and mothers as a dyad, and did not look for gender differences (Tait and Mundia, 2012; Vetrayan et al., 2013).

In further regards to gender differences in parenting stress among parents of children with ASD, this systematic review was able to identify some possible trends that may be specific to mothers and fathers respectively. For example, in an early study (Liwag, 1989), mothers were found to express stress in respect to the symptoms and disabilities associated with autism itself and worries about if they were not alive to care for the child; whereas, fathers emphasized fears that the child will never be “normal” and anxiety that the child will always be dependent and that the family may not be able to meet the child's needs (Liwag, 1989). In a more recent study, Resurreccion (2013) found mothers to highlight the importance of “parental involvement” and caregiver responsibilities in her child's development, whereby half of the mothers sampled in the study were found to resign from their work to become full-time mothers in assisting their child at home (p. 104); as did some of the mothers in the study by Foronda (2000). On the other hand, fathers were found to bear the responsibility of meeting the educational, health, and financial needs of the child as well as of the family (e.g., Liwag, 1989; Resurreccion, 2013). Together, these findings initially suggest that whilst a nurturing/emotional theme possibly underlies the factors that affect the stress experienced by mothers, fathers may be more likely to respond to stress based on their role as a provider in the family.

In parallel to this, findings from quantitative studies (Nikmat et al., 2008; Rejani and Ting, 2015) also provide support to the notion of possible gender differences in how parents of children with ASD experience stress. Particularly in the study, mothers' higher rates of psychological distress (i.e., stress, anxiety, and depression) were found despite an absence of gender differences in level of parenting stress (Nikmat et al., 2008). Here, it is important to note that whilst “stress” is a sub-component of psychological distress, it is compounded with other variables such as depression and anxiety in the conceptualization of psychological distress. As such, this finding (Nikmat et al., 2008) further corroborates the idea that whilst both mothers and fathers of children with ASD experience parenting stress, there may be an “emotion-focused” theme that underlies more strongly maternal than paternal stress. However, this observation is limited to the small number of studies that compared mothers and fathers and the comparatively fewer number of fathers who were recruited as participants. Therefore, further research in respect to these observations are needed to provide better insight and understanding of how parenting stress is differentially experienced by mothers and fathers in the SEA region, and thus, how intervention methods can be tailored to meet these differences.

Additionally, several studies (Rahman et al., 2012; Ha et al., 2014; Ilias et al., 2016) have found cultural beliefs (e.g., karma, parental sins, curses) to influence how society perceives the diagnosis of ASD, which is less commonly found in studies conducted in a Western context. Parents who are persuaded by such beliefs may adopt an external locus of control, such that they feel helpless and adopt maladaptive methods (e.g., substance abuse) to cope with the stress experienced as found in the study by Rahman et al. (2012). However, as observed through the current systematic review, a greater number of studies (Liwag, 1989; Foronda, 2000; Wahyuni, 2013; Xue et al., 2014; Quilendrino et al., 2015; Siah and Tan, 2015, 2016; Chong and Kua, 2016; Ilias et al., 2016) found parents to adopt a positive perception and understanding toward ASD (e.g., adaptability, emotional acceptance, positive belief, sense of coherence) and hence, effectively cope with the obstacles of raising a child with ASD. It is therefore plausible, that the South-East Asian society is beginning to move away from cultural beliefs of parental sins, karma and/or curses in the etiological understanding of the ASD diagnosis.

Two other factors (i.e., parents' anxiety and worries about child's future, and religious belief) were also found to be associated with parenting stress among parents of children with ASD. In regards to the parents' anxiety and worries about the child's future, this factor was related to the lack of resources and support services available in the region to manage the ASD symptomologies and associated comorbidities (Neik et al., 2014). This finding suggested that parents in the SEA region face an imperative need for more resources and support services. Despite the inconsistency in the presence or absence of both these factors across the reviewed studies, these factors have also been found elsewhere (e.g., Ooi et al., 2016). However, in this paper, “religious belief” stood out as a culturally related factor as it was comparatively more salient among the reviewed studies (i.e., in the SEA region) than in studies conducted in a Western/European context (e.g., Hayes and Watson, 2013; Ooi et al., 2016). However, even in other contexts, there is evidence suggesting that the role of religion may serve as a positive source of support and aid in the initial acceptance and accommodating phase of adjustment (e.g., Tarakeshwar and Pargament, 2001; Gupta and Singhal, 2004; Benson, 2010; Willis et al., 2016).

Furthermore, although the age of a child with ASD is a variable that has typically been found to affect parenting stress among parents of children with ASD in a Western context (e.g., Duarte et al., 2005; Tehee et al., 2009; Rivard et al., 2014), it was not highlighted across the studies in this review except for one study (Yeo and Lu, 2012), which found parenting stress (and its relationship to psychological distress) to differ between mothers of preschool and elementary children with ASD in Malaysia and China. The study also investigated the age of a child with ASD as a variable, though not in respect to parenting stress (Vetrayan et al., 2013). Instead, authors found no correlation between the age of the child with parents' level of hopelessness (Vetrayan et al., 2013). The scarcity in report of this factor in this review may not be due to the lack of its implications in parenting stress. Rather, it may more accurately be attributed to the aims and designs of the studies in this review that were more interested in the analyses of other variables in relation to parenting stress. Or possibly, age was measured and analyzed but was not included in a particular study due to its non-significant findings and/or space constraints in the publication of the research.

On the whole, this systemic review is limited such that is it restricted to the findings of the studies reviewed in this article. Notwithstanding a thorough search of the literature review in multiple databases, only 28 studies related to parenting stress among parents of children with ASD in the SEA region was yielded. Whilst there is a possibility that selection bias occurred, this is less likely to be case as the authors were cautious to include studies that investigated other variables (e.g., hopelessness, parental satisfaction, quality of life) that might also provide a better understanding of the parenting stress experienced by parents of children with ASD. Hence, the limited number of studies that met the inclusion criteria despite this precautionary step might also point to the lack of research conducted in the SEA region in regards to parenting stress and resilience among parents of children with ASD.

At face value, it is noticeable that low-income countries and those with lower ratings in the Human Developmental Index (United Nations Development Programme, 2013) in the SEA region were underrepresented in this systematic review; specifically, Cambodia, East Timor, Laos, and Myanmar. It is thus possible that the findings are biased toward countries in the region with higher ratings in the Human Developmental Index. Accordingly, this necessitates the advancement and expansion of the research area on ASD in this region, especially in regards to parenting stress and resilience. It should be pointed out, that in the SEA region, where native, traditional languages (e.g., Burmese, Chinese, Cambodian, Indonesian, Lao, Malay, Tagalog, Thai, and Vietnamese) are still spoken among the people, research articles published in these languages and in relation to parenting stress could illuminate on factors that may have been overlooked in the English-only, published articles included in this review. Nevertheless, very few articles were identified in native languages, although initial attempts were made to locate them as well. The English-only search strategy could have introduced bias leading articles published in local languages to be overlooked. Future reviews are recommended to include multi-languages in the search process.

Secondly, the overrepresentation of mothers/ underrepresentation of fathers in this paper might lead to a gender bias in the reported findings. The large difference in the number of mothers and fathers sampled across studies in this review paper may reflect the easier nature to recruit mothers in comparison to fathers as they, in general, remain to be the primary caregiver in an Asian context (Phetrasuwan and Shandor Miles, 2009; Rejani and Ting, 2015; Siah and Tan, 2015). On the other hand, this could also be attributed to the sampling methods that aim to recruit only one parent of a child with ASD (usually the mother) rather than both parents. As elaborated above, this makes it difficult to thoroughly determine how parenting stress is experienced differently for mothers and fathers. This overrepresentation of mothers has also been a commonly observed bias in Western samples (e.g., Bitsika et al., 2013; Braunstein et al., 2013; Hayes and Watson, 2013; Ooi et al., 2016).

Moreover, majority of the studies in this review did not also report the age at which the children were diagnosed with ASD. Whilst the children of some parents would have received an earlier diagnosis, other children might have only just been diagnosed relative to the time at which the respective study was conducted. This difference might lead to parents of children with an earlier diagnosis, to over time, be more equipped and resilient in caring for their child with ASD in comparison to parents who are only beginning to seek for clarity and support. As such, if this factor was instead maintained as a control variable, it would have allowed for a clearer and more accurate examination of parenting stress among parents of children with ASD.

Further limiting the conclusions made in this review, the common use of purposeful and convenience sampling may have led to a biased sample of more resilient parents who are more likely to volunteer and participate (e.g., Tait and Mundia, 2012; Wisessathorn et al., 2013; Xue et al., 2014; Chong and Kua, 2016; Ilias et al., 2016). Even, in one article (Foronda, 2000), the author was transparent reporting she included herself as one of the 16 participants. No other conflicts of interest were specifically reported, although it is possible they may have been present in some studies. Other than that, the sampling could have also led to a biased sample of more parents from middle to higher socioeconomic backgrounds who have access to intervention services (e.g., Liwag, 1989; Sian and Tan, 2012; Ha et al., 2014; Xue et al., 2014; Santoso et al., 2015; Siah and Tan, 2016). Additionally, studies included in the review were cross-sectional and therefore inferred an association rather than a causal relationship. Longitudinal investigations are recommended for future research.

Besides that, variation in designs, factors analyzed, methods, and outcome measures, made direct comparison between the findings of the studies, problematic. As illustrated in Supplementary Table 1, significant variation was observed for the outcome measures of parenting stress, and conceptual ambiguity of the construct remains an area for future investigation to tackle. Furthermore, the use of an active control group in only one of the reviewed studies (Lai et al., 2015) elucidates the need for more experimental/quasi-experimental design components in the research area of ASD in the SEA region. The 28 studies had a reasonable balance between quantitative and qualitative studies (14 quantitative, 11 qualitative, and three mixed-method) that were found to corroborate the findings among them. Therefore, as a seminal article (at least, to our knowledge) that has reviewed articles in the SEA region in regards to parenting stress among parents of children with ASD, this paper provides a helpful and informative starting point for future researchers, parents, as well as professionals in the field.

To conclude, this systematic review observed four main factors to be associated with parenting stress among parents of children with ASD in the SEA region: social support, severity of autism symptoms, financial difficulty and parents' perception and understanding toward autism. Other factors that were also found to be associated with parenting stress in this sample (though, to a lesser extent) included, parents' anxiety and worries about their child's future, and religious belief. Whilst several of these factors may have similarly been reported in a Western context, findings from the current systematic review lean to suggest that the greater advanced development and implementation of policy, economical and professional support services in Western countries than in South-East Asian countries might impart cultural differences in regards to how these factors are manifested. Therefore, further research is imperative to more clearly identify cultural specificities that differentiate how parenting stress and its associated factors are uniquely experienced across countries in the SEA region in comparison to Western countries. Additionally, as majority of the studies identified in this review were not published in high-impact journals with large readerships, funding and research bodies are recommended to increasingly support the methodological rigor in the design, conduct, and publication of studies in the SEA region, especially in the lower-income countries.

## Author contributions

All authors listed have made a substantial, direct and intellectual contribution to the work, and approved it for publication.

### Conflict of interest statement

The authors declare that the research was conducted in the absence of any commercial or financial relationships that could be construed as a potential conflict of interest.
