# Supplementary material for: Parenting Stress and Resilience in Parents of Children With Autism Spectrum Disorder (ASD) in Southeast Asia: A Systematic Review
Source: Front Psychol. 2018 Apr 9;9:280. doi: 10.3389/fpsyg.2018.00280 (PMC5900388; doi:10.3389/fpsyg.2018.00280)
Supplement: Supplementary file 1 [file Table1.pdf]

Supplementary Table 1. Studies included in this Systematic Review

Study	Location	Design	Sample	Aim/Goal	Outcome Measures	Findings
Athari, Ghaedi, and Kosnin (2013)	Malaysia	Quantitative study.	250 mothers of children with autistic disorder, aged six to eight, were recruited from special schools and clinics.	To investigate the relationship between levels of mothers' depression and stress with severity of autism symptoms in their child, and if both these variables are affected by family income.	Mother's level of depression and stress were measured using the Depression, Anxiety and Stress Scale (DASS-42; Lovibond and Lovibond, 1995).<br><br>Severity of autism was assessed using teacher's report on the Autism Behavior Checklist (ABC; Krug, Arick, and Almond, 1980).<br><br>A self-report questionnaire was administered to determine family income, without considering the proportion of income between mothers and fathers.	Level of depression and stress amongst mothers of children with autistic disorder were found to significantly and positively correlate with severity of autism diagnosis, and this relationship was mediated by family income.<br><br>As a stand-alone variable, family income had a significant, negative correlation with both level of mothers' depression and stress, and severity of autism respectively.<br><br>Also, regression coefficients indicate that level of depression and stress amongst mothers of children with autistic disorder could significantly predict the severity of autism among their child.

Study	Location	Design	Sample	Aim/Goal	Outcome Measures	Findings
Callos (2012)	Philippines	Quantitative study	20 parents (17 mothers, 3 fathers) of children with ASD, who were enrolled in Gen. Maximino Hizon Elementary School, Manila, Philippines.	To study the relationship between demographic variables among parents of children with ASD (i.e., age, gender, occupation, number of children, and educational attainment) with their level of stress and their coping mechanisms.	The Questionnaire on Resources and Stress (QRS-F; Friedrich, Greenberg, and Crnic, 1983) was used to measure parenting stress.<br><br>The Coping Health Inventory for Parents (CHIPs; McCubbin et al., 1983) was used to measure coping mechanisms, which included three subscales, Maintaining Family Integration, Cooperation and an Optimistic Definition of the Situation (MFI); Maintaining Social Support, Self Esteem and Psychological Stability (MSS); and Understanding the Medical Situation through Communication with Other Parents and Consultants (UMS).	Overall levels of parenting stress were found to be medium, suggesting parents have somewhat adapted to their child's situation. Stress levels did not significantly differ according to parents' demographic variables.<br><br>No significant differences were also found for the effectiveness of coping mechanisms as described within the three subscales of the CHIPs, when parents were grouped based on demographic variables.<br><br>There was a significant negative relationship between parents' level of stress and the coping mechanism described under the subscale MFI.<br><br>Future studies were encouraged to examine the level of stress experienced by fathers, and to therefore determine the parent who is more affected by the child's diagnosis and who has better coping mechanisms.

Study	Location	Design	Sample	Aim/Goal	Outcome Measures	Findings
Charnsil and Bathia (2010)	Thailand	Quantitative study.	27 caregivers (24 parents, 3 relatives; 15 females, 12 males), of children diagnosed with autistic disorder as according to the DSM– IV–TR (APA, 2000) criteria, and who spoke fluent Thai, were recruited from the Outpatient Clinic of the Child Psychiatric Unit, Maharaj Hospital, Chiang Mai, Thailand.	To determine the prevalence of depression among caregivers of children with autistic disorder, and the factors associated with it.	The Mini International Neuropsychiatric Interview (MINI) Thai version 5.0 Models A and B were used to diagnose the caregivers as with or without clinical depression.<br><br>The Childhood Autism Rating Scale (CARS; Schopler, Reichler, DeVellis, and Daly, 1980) was used to measure severity of autistic disorder in the child.	The prevalence of clinical depression in this sample was 25.6% (n = 7), which was suggested to be higher than the general population in Thailand.<br><br>Only number of years of education was found to significantly predict clinical depression in the caregivers.<br><br>Severity of autistic disorder was reported to be higher among children of depressed caregivers than non-depressed caregivers; though, this difference was reported to be only marginally significant.<br><br>The lack of correlation between other factors (e.g., gender of parents, age of parents, marital status amount of time in child care) and depression was suggested to likely be due to the protective power of the strong social support received by extended family members, friends and community that are typically seen in the collective and supportive Thai society.

Study	Location	Design	Sample	Aim/Goal	Outcome Measures	Findings
Chong and Kua (2016)	Singapore	Qualitative study.	10 mothers of children with autistic disorder, who were attending a special needs school in Singapore.	To determine how mothers of children with autistic disorder foster their parenting self-efficacy.<br><br>To identify additional sources of parenting self-efficacy.<br><br>To understand how these mothers manage their negative experiences.	Nil.	Mastery experiences were identified as the most critical source of parental self-efficacy, with physiological and affective states being secondary.<br><br>Support received from various groups (e.g., spouse, family members, friends, parent support groups, work superiors, school teachers, mental health professionals) significantly fostered mothers' perceived capability.<br><br>Three mothers reported their religious belief to be a source of hope that helped them through their struggles.<br><br>Mothers were also found to frame their negative experiences within a positive mindset (i.e., problem-solving capabilities, to perceive challenges as a learning process, accepting their child's diagnosis, acknowledging that their child have different needs, reflecting and

Study	Location	Design	Sample	Aim/Goal	Outcome Measures	Findings
						learning from past experiences) to boost their perceived capability.
Foo, Yap, and Sung (2014)	Singapore	Qualitative study.	6 parents (3 mothers, 3 fathers) who met the following inclusion criteria:<br>(i) Participant's child had a clinical diagnosis of ASD with challenging behaviours.<br>(ii) Participants had to be English-speaking.<br>(iii) Participant's child was in the age range of 6–18 years old.<br>(iv) Child's Total Problems T score on the Child Behaviour Checklist for Ages 6–18 (CBCL/6–18; Achenbach and Rescorla, 2001) had to meet 60 and above.	To explore the lived experience of parent-caregivers in Singapore with a child diagnosed with ASD, and how these parents make sense of their experiences.	Nil.	Two dominant themes were found to emerge from the analysed transcripts: sense of responsibility (parental, spousal and community duty) and renewed self-appreciation (i.e., in relationship with their child, spouse, and social environment, parents found appreciation of personal strengths and resourcefulness).<br><br>Four other themes that were found (though not as prominent as the aforementioned themes) are: psychological experiences that change over time, psychological experiences that remain over time, behavioural responses and relational experiences.<br><br>Parents were reported to advocate for additional resources and counseling services for families of children with ASD. Government support and funding were suggested as key contributors in aiding service providers provide support for parents.

Study	Location	Design	Sample	Aim/Goal	Outcome Measures	Findings
Foronda (2000)	Philippines	Mixed method.	16 single mothers (primary caregivers) of children with ASD, who were referred by principals of special schools in Manila and Laguna. The study author was also included as a participant.	To determine the types of stressors encountered by single mothers of children with ASD, and their associated coping strategies.	The integration of quantitative self-administered questionnaires and qualitative methods via: self-administered questionnaire, semi-structured interview, and non-obtrusive observation. Three case studies representing different levels of coping by the single mothers were also discussed.	The respondents described their sources of stress to be related to ASD symptoms exhibited by their child, financial difficulties, anxiety about the future, excessive demand on time, lack of autism awareness within the community and having to take on several roles.<br><br>The study found these single mothers to cope with stress, by: (i) holding positive beliefs on having a child with ASD (i.e., expressing love, pride and commitment towards the child, attitude towards autism, and support system), (ii) drawing from family support (mostly from their spouse, and least from their middle children and spouses' relatives) and (iii) by relying on informal (e.g., relatives, friends, work colleagues, neighbours, church members, and other parents of children with ASD) and formal (e.g., special school, speech and physical therapy, and parent training and

Study	Location	Design	Sample	Aim/Goal	Outcome Measures	Findings
						psychological counseling) social support.<br><br>Greater support services, research, and policy and legislation are proposed.
Ilias, Liaw, Cornish, Park, and Golden (2016)	Malaysia	Qualitative study.	8 mothers of children with autistic disorder who were recruited with the distribution of flyers at treatment centres, schools and online ASD support groups.	To explore how mothers found meaning in their experiences of raising a child with autistic disorder.	Nil.	Three themes were found to underlie how these mothers adapted and cultivated positive well-being: (i) the diagnostic process of identifying autism and its related challenges in Malaysia, (ii) how mothers gave meaning to their experiences in raising a child with ASD, and (iii) the coping and adaptation process of that fosters well-being in these women.<br><br>Theme 1 included: limited awareness of ASD in Malaysia, cultural beliefs on ASD, lack of governmental resources, and worries of the child's future.<br><br>Challenges/support within Theme 2 included: family quarrels, mothers as the primary caretaker, father's denial and acceptance process, nuclear family support, supportive extended family, daily

Study	Location	Design	Sample	Aim/Goal	Outcome Measures	Findings
						challenges of autism symptoms, reduced social life, and mixed emotions.<br><br>Theme 3 included: a more resilient character, proactive mindset, acceptance and purpose, shifting parental expectations, spiritual beliefs and parental support networks.
Ha, Whittaker, Whittaker, and Rodger (2014)	Vietnam	Qualitative study.	27 parents (21 mothers, 6 fathers), who were recruited from the Hanoi Club of Parents of Children with ASD, clinics, special schools, and via the snowball technique.<br><br>17 key informants were also interviewed (i.e., managers of intervention centres, service providers and policy makers).	To determine how ASD is represented in Hanoi, Vietnam; and to describe the experiences of families of children with ASD in Hanoi, Vietnam.	Nil.	Main themes found from parents' responses included: perceptions of disability and ASD in Vietnam, fears of their child's uncertain future, discrimination faced by their children with ASD, assessment and diagnosis services, intervention services, and syncretic care.<br><br>Overall, families of children with ASD in Hanoi experienced stigma and discrimination. Parents also experienced challenges in the assessment and diagnosis of ASD in their children, and had limited access to treatment services for their children. The poor political and economic support further strained the family. Parents

Study	Location	Design	Sample	Aim/Goal	Outcome Measures	Findings
						reported very limited support from professionals and the government in assessment and intervention services for their children.
Lai, Goh, Oei, and Sung (2015)	Singapore	Quantitative study.	136 parents (110 mothers, 26 fathers; 73 parents of children with ASD and 63 parents of children with typical development).	To examine the differences in psychological well-being and coping between parents of children with ASD, and those with children of typical development.	A Demographic Screening Form was used to obtain participants' responses on demographics that were assumed to impact parent outcomes and coping.<br><br>Parental stress was measured using the Parenting Stress Index: Short Form (PSI-SF; Abidin, 1992).<br><br>Psychological well-being was measured using the Depression, Anxiety and Stress Scale (DASS-21; Lovibond and Lovibond, 1995).	In comparison to parents of children with typical development, parents of children with ASD were reported to experience significantly greater symptoms of parental stress (i.e., negative parental self-views, lower satisfaction with parent–child bond, and experiences of difficult child behaviours), greater depression symptoms and more frequently adopted the use of maladaptive coping strategies (i.e., Active Avoidance).<br><br>No significant differences in parenting stress, psychological well-being and coping were found among parents, based on their child's ASD diagnosis (i.e., Autism, Asperger's and PDD-NOS).

Study	Location	Design	Sample	Aim/Goal	Outcome Measures	Findings
					Coping strategies were measured via the Brief COPE (Carver et al., 1989), which analyses four sub-domains: Active avoidance coping, problem focused coping, positive coping, and religious/denial coping.	
Liwag (1989)	Philippines	Qualitative study	13 families (13 mothers, 12 fathers) who had at least one child who has been diagnosed with autism as according to the Diagnostic and Statistical Manual of Mental Disorders (3rd ed.; DSM-III; American Psychiatric Association, 1980) criteria, and who were recruited from a private clinic in Manila and from eight special schools.	To explore the stress and coping mechanisms amongst families with children diagnosed with autism, and how these experiences may differ for mothers vs. fathers.	The study was conducted in two phases, whereby in Phase 1, parents were asked to fill up a questionnaire on familial demographic characteristics and parental attributes. In Phase 2, a Sentence Completion Form (SCF) and in-depth interviews were used to analyse parents' emotional responses and attitudes towards their child with autism.<br><br>However, only findings from the SCF	Content analysis on the SCF suggested families (mostly mothers) to be most stressed due to the disabilities associated with autism, which includes lack of speech, hyperactivity and behavioural tantrums. Mothers additionally stressed about the prospect of their child, should they (the mothers) no longer be around.<br><br>A second source of stress that was found (mostly by fathers) was the permanence of the child's diagnosis, compounded with the anxiety that their child will never be normal or independent. Fathers were also stressed out with the anxiety that they might not be able to meet the special requirements of

Study	Location	Design	Sample	Aim/Goal	Outcome Measures	Findings
					were reported in the paper.	their child (e.g., education, health, finance).<br><br>Two forms of coping strategies were identified: instrumental adjustments (i.e., time, effort, attention and expenses are focused on the needs of the child with ASD, such as seeking appropriate professional assistance – therapy, medication, school -, learning techniques for effective daily management of the child and incorporating parental intervention programs as part of the child's treatment) and emotional acceptance (i.e., to be extra patient and understanding towards the child with autism).<br><br>Difference between mothers and fathers were also found, such that more mothers reported coping efforts that required having to make more sacrifices, whereas more fathers reported coping efforts in terms of exerting greater effort in attending to the needs of the child.

Study	Location	Design	Sample	Aim/Goal	Outcome Measures	Findings
						Support from extended family members, professional support and stable finance were other factors that were reported to contribute to the families' coping efforts.
Moh and Magiati (2012)	Singapore	Quantitative study.	102 parents (85 mothers, 17 fathers).<br><br>17 professionals providing ASD diagnostic services.	To determine how parental satisfaction and stress during the autism diagnostic period is affected by: duration of diagnostic period, number of professionals consulted, relationship with the professionals, and perceived helpfulness of information received.	The survey distributed to parents included a number of items from Howlin and Moore (1997), which asks for child's age at diagnosis, type and number of professionals consulted, and type and perceived importance of possible information received, which was then used to calculate the Helpfulness of Information Index.<br><br>The Family-Professional Collaboration Scale (FPCS; DeChillo, Koren and Schultze, 1994) was used to measure the relationship between	Higher parental stress was associated with greater number of professionals consulted and lower perceived collaboration with the professionals, but not with autism symptom severity.<br><br>Higher parental satisfaction was associated with higher perceived collaboration with the professionals, higher perceived helpfulness of information received, lower severity of ASD symptoms and lower levels of stress.<br><br>Collaboration with professionals, perceived helpfulness of information provided and parental stress were significant contributors of parental satisfaction. Autism severity did not significantly predict parental satisfaction.

Study	Location	Design	Sample	Aim/Goal	Outcome Measures	Findings
					parents and professional(s).<br><br>By asking parents to provide a retrospective rating of the stress experienced in regard to four aspects of the diagnostic process (i.e., waiting time, how the diagnosis was communicated, number of referrals, communication with referrals) a Parental Stress Index and a Parent Satisfaction Index were calculated.<br><br>Autism severity and current functioning of the child with ASD, was measured using the Autism Treatment Evaluation Checklist (ATEC; Rimland and Edelson, 2000).	Whilst number of professionals consulted and perceived collaboration with professionals were found to negatively correlate with parental stress, these factors however, were not found to explain a large amount of variance in predicting parental stress (i.e., 8%). This was assumed to be due to the lack of accuracy in a retrospective rating of stress in comparison to the actual stress experienced. It was also suggested that aspects of the diagnostic process (i.e., number of professionals consulted, collaboration with professionals, helpfulness of information) might indirectly affect parental stress by affecting levels of parental satisfaction.
Nikmat, Ahmad, Oon,	Malaysia	Quantitative study.	52 parents of children with ASD (34 mothers, 18	To determine the prevalence of parental stress and	Psychological wellbeing, parental stress and dimensions	Findings from this study show 90.4% of the sampled parents experience parental stress, whereas

Study	Location	Design	Sample	Aim/Goal	Outcome Measures	Findings
and Razali (2008).			fathers, who were randomly selected from a psycho-education session on managing children with autism.<br><br>Exclusion criteria included parents with a history of psychological disorders (i.e., depression and/or anxiety) and/or having any general medical condition that could impact the study.	psychological well-being amongst parents of children with ASD, and its relationship with dimensions of social support received.	of support system were assessed using the General Health Questionnaire (GHQ-28; Goldberg, 1978), the Parenting Stress Index (PSI; Abidin, 1992) and the Provision Social Relation (PSR; Turner, Frankel, and Levin, 1983) respectively.	53.8% of them showed clinical disturbance in their psychological well-being.<br><br>Besides parents' occupation, gender of parent was also found to significantly affect parents' psychological well-being, whereby, mothers report higher psychological distress in comparison to fathers. However, there were no gender differences in terms of parental stress.<br><br>On the other hand, all participants were found to report dysfunctional parent-child interaction as a significant source of parental stress, followed by difficult behaviours of the child, and parental distress (i.e., distress experienced about their parenting role in respect to other personal stress).<br><br>No significant associations were found between other demographic variables (i.e., parental age, ethnicity, housing area, occupation, total household income and education level), dimensions of

Study	Location	Design	Sample	Aim/Goal	Outcome Measures	Findings
						social support received, parental stress, and psychological wellbeing.<br><br>Also, perceived severity of autism symptoms was found to not significantly correlate with parental stress, psychological wellbeing and dimensions of support system received.
Quilendrino, Castor, Mendoza, Vea, and Castillo-Carandang (2015)	Philippines	Mixed Method, with two phases. (Phase 1: Key Informant Interviews and Focus Group Discussions; Phase 2: Survey based on questionnaire generated from Phase 1).	15 parents (9 mothers, 6 fathers) in Phase 1; 41 parents (34 mothers, 7 fathers) in Phase 2.<br><br>Parents were recruited from parent organizations, special schools, therapy centres, and clinics.	To determine parents' perceptions of autism and their health – seeking behaviours.	Nil.	No significant associations were found between baseline characteristics of parents and children with parental perceptions of autism; nor between parental perceptions of autism and their health-seeking behaviours.<br><br>Parents' perceptions of autism:<br>- Most parents were in denial and hoped their child will improve in development.<br>- Majority of the parents feared for their child's future and care.<br>- Majority of the parents have a general idea that genes and the environment play a role in the etiology of autism.<br>- Generally, parents value the opinions of relatives and friends,

Study	Location	Design	Sample	Aim/Goal	Outcome Measures	Findings
						although majority reported straight to a professional.<br><br>Parents’ health-seeking behaviour:<br>- Majority of the parents in the focus groups sought the opinion of general pediatricians for their child’s autism symptoms.<br>- Several parents were unable to meet right away with a professional due to insufficient finance, lack of knowledge, long waiting time to schedule an appointment.<br>- Parents agreed that factors such as parental sins and curses were unrelated to the diagnoses of their child with autism.<br>- There was a trend for earlier initial consult, implying heightened awareness among parents and society as well as better diagnostic services in recent years in comparison to the past.<br>- Parents with younger children were more likely to have earlier symptom recognition and diagnoses of autism.

Study	Location	Design	Sample	Aim/Goal	Outcome Measures	Findings
Rahman, Ismail, Jaafar, Fong, Sharip, and Midin (2012)	Malaysia	A qualitative, case study.	One couple (a father and a mother).	To highlight the factors related to a family faced with discrimination against special children, and the challenges faced by service providers in managing a family caring for children with developmental disorders (ASD).	Nil.	Both father and mother were diagnosed with depression and contemplated suicide.<br><br>The mother did not receive support from her family, while the father was rejected by his.<br><br>Factors that could have contributed to the suicide ideation include social isolation, dependence on one another, unemployment, serious illness and threat of being separated from each other.<br><br>Parents faced barriers in receiving services (i.e., needing to fulfill basic needs first, cultural and language differences, appointment timing, transport, finances).<br><br>Suicidal ideation subsided after getting support from psychiatric services – a fully supportive family-centred and culturally–sensitive service.
Rejani and Ting (2015)	Malaysia	Quantitative study.	20 parents (10 mothers, 10 fathers), who were recruited from a day	To determine the level of depression and anxiety among	Levels of depression were measured using the Beck Depression Inventory (BDI; Beck,	Mothers were found to experience higher levels of depression and anxiety in comparison to fathers.

Study	Location	Design	Sample	Aim/Goal	Outcome Measures	Findings
			care centre for children with autism in Sarawak, Malaysia.	parents of children with ASD.	Steer, and Brown, 1996).<br><br>Levels of anxiety were measured using the State-Trait Anxiety Inventory for Adults (STAIS-AD; Speilberger, 1983).	None of the fathers experienced depression, though majority of the fathers experienced mild levels of anxiety.
Resurreccion (2013)	Philippines	Qualitative study, whereby data collected was analysed using he Consensual Qualitative Analysis (CQR; Hill et al., 2005).	10 couples (10 fathers, 10 mothers) of children with autistic disorder, who were recruited mainly via referrals or from the community based rehabilitation centres.	To determine the best practices (i.e., coping strategies) of parents of children with autistic disorder, as well as that of the interventionists of their child.	Nil.	Results obtained found coping strategies acquired by both fathers and mothers to be instrumental (i.e., ‘acquiring knowledge on autism’) and emotional (i.e., ‘providing unconditional love’).<br><br>Fathers further added the need to “identify the appropriate intervention” for their child, whilst mothers stressed the importance of “parental involvement” with their child’s development whilst concurrently striking a balance in looking out for their own health by taking time out of maternal duties.<br><br>Both parents were also found to be appreciative of interventionists who provided “supportive

Study	Location	Design	Sample	Aim/Goal	Outcome Measures	Findings
						guidance" (i.e., giving essential information) and "personal care" for their child (i.e., implements personalised programs and is compassionate towards the child).
Roffeei, Abdullah and Basar (2015)	Malaysia	Qualitative study.	A total of 3637 messages (381 postings and 3256 comments) were collected from August to November 2013 from two online autism support groups on Facebook (i.e., Autism Malaysia and Autism Children Club).	To examine the type of social support messages that are exchanged among parents and caregivers of children with ASD on online Facebook groups.	Social support themes identified in the messages (qualitative data) were adapted from the Social Support Behavior Code (SSBC; Cutrona and Suhr, 1992).	Two types of support messages were exchanged most frequently on online communities of Facebook autism groups: Informational Support, followed by Emotional Support.<br><br>Messages exchanges demonstrated a strong support from parents and relatives of children with ASD, in comparison to the roles of other users.<br><br>Majority of the messages were centred on the challenges in raising a child with ASD, including issues such as their child's social lives and self- care routines.
Santoso, Ito, Ohshima, Hidaka, and Bontje (2015)	Indonesia	Qualitative study using two focus groups, where a constant	14 mothers of children with ASD, aged 18 years or younger.	To determine and describe the processes influencing resilience amongst Indonesian	An eight-question interview guide was used for both focus groups during the first session; also during which, a series of new	A model of resilience was found, which entailed the following four categories: (i) creating and re-creating accepting conditions, (ii) finding solutions, (iii) striving for balance among daily occupations,

Study	Location	Design	Sample	Aim/Goal	Outcome Measures	Findings
		comparative method was used for data analysis.	Inclusion criteria:<br>(i) mother of a child with ASD who is aged 18 years or younger, (ii) resides in Jakarta, Indonesia and (iii) can speak Indonesian.	mothers of children with ASD, within the context of their daily occupations.	questions were formulated for the second session.	and (iv) thinking about the child's future.<br><br>Creating and Re-creating accepting conditions:<br>- The foundation of participants' resilience.<br>- This was highly influenced by support from family members, especially the spouse and other people involved with the child (e.g., teachers).<br><br>Finding solutions:<br>- Mothers reported gaining inner strength by participating in religious practices.<br>- External sources such as, professional support and educational seminars were also sought after.<br>- Mothers also reported prioritizing household duties as a strategy.<br><br>Striving for balance among daily occupations:<br>- Daily plans were helpful to mothers in ensuring the smooth running of their daily occupations.<br><br>Thinking about the child's future:

Study	Location	Design	Sample	Aim/Goal	Outcome Measures	Findings
						- Thinking about their child's future was seen as a motivator and thus, a sign of resilience among mothers.<br>- Mothers' thoughts of their child's future included hopes that their child would be independent, get a job, raise a family, and etc.
Siah and Tan (2015)	Malaysia	Quantitative study	96 parents (72 mothers, 24 fathers) of children with ASD, who were recruited from three non-governmental organisations (NGOs) in Malaysia.	To determine the relationship between sense of coherence and quality of life among parents of children with ASD.	Quality of life was measured using the The World Health Organization Quality of Life Schedule Brief version (WHOQoL – BRIEF; Group, 1998), which is categorised into four subsets: physical health, psychological health, social health and environmental domain.<br><br>The Sense of Coherence scale (Antonovsky, 1987) contains 13 items, which measures three factors: (i) comprehensibility (the extent to which events	Parents identified as having higher sense of coherence had higher quality of life than those identified with lower sense of coherence.<br><br>Also, not all types of sense of coherence were relevant to quality of life:<br>- comprehensibility was relevant to all four QoL.<br>- manageability was relevant to physical and psychological health.<br>-meaningfulness was not relevant to any QoL domains.

Study	Location	Design	Sample	Aim/Goal	Outcome Measures	Findings
					are perceived as making sense), (ii) meaningfulness (the sense that challenges faced are worthwhile and (iii) manageability (the feeling of being capable to respond to situational demands).	
Siah and Tan (2016)	Malaysia	Quantitative study.	92 parents of children with ASD (69 mothers, 23 fathers) were recruited from 3 NGOs in Malaysia.	To determine the relationships between sense of coherence, coping strategies and quality of life among parents of children with ASD.	Parents' quality of life was measured using The World Health Organization Quality of Life Schedule Brief version (WHOQOL-BREF; Group, 1998), which constitutes of four subscales: physical health, psychological health, social relationships and environmental factor.<br><br>Parents' sense of coherence was measured using the Sense of Coherence scale (Antonovsky, 1987).	Parents' sense of coherence was found to correlate with all four domains parents' quality of life, and was negatively correlated with two coping strategies (i.e., distraction and disengagement).<br><br>"Cognitive reframing" was positively correlated with "psychological health".<br><br>"Disengagement" was negatively correlated with "psychological health," "social relationships," and "environmental factors."<br><br>"Distraction" was negatively correlated with "social relationships" and environmental factors".

Study	Location	Design	Sample	Aim/Goal	Outcome Measures	Findings
					Parents' coping strategies were measured using the Brief COPE (Carver, 1997), which has a total of 28-items to measure: "engagement," "distraction," "disengagement," and "cognitive reframing."	"Engagement" did not significantly correlate with any subsets of parents' quality of life.<br><br>Parents' sense of coherence and "cognitive reframing" were found to predict parents' quality of life.<br><br>None of parents' demographic (e.g., age, gender, employment, education) were found to predict their quality of life.<br><br>Policy makers and service providers were encouraged to organise more workshops for parents of children with ASD.
Sian and Tan (2012)	Malaysia	Quantitative study.	47 parents (36 mothers, 11 fathers) of children with ASD were recruited from 3 NGOs in Malaysia.	To investigate the relationship between situational motivation and quality of life amongst parents of children with ASD in Malaysia.	Situational motivation (SIMS; Guay, Vallerand, and Blanchard, 2000) measures four types of motivation: intrinsic motivation, identified regulation, external regulation, and amotivation.<br><br>Parents' quality of life was measured using	Significant associations found in this study suggested that parents with higher intrinsic motivation to participate in programs (developed to assist parents in coping with stress, and to improve their QoL) have better social relationships (a dimension of QoL) in comparison to parents with lower intrinsic motivation.<br><br>Also, parents' who scored higher on 'identified regulation'

Study	Location	Design	Sample	Aim/Goal	Outcome Measures	Findings
					The World Health Organization Quality of Life Schedule Brief version (WHOQOL-BREF; Group, 1998), which constitutes of four subscales: physical health, psychological health, social relationships and environmental factor.	experienced significantly better physical health compared to those who scored lower on 'identified regulation.'<br><br>No other significant associations were found.
Tait and Mundia (2012)	Brunei	Mixed method.	40 parents (30 mothers, 10 fathers) from 30 Bruneian families, of children with ASD, who were attending the SMARTER Centre in Bandar Seri Begawan, Brunei.	To investigate parents' perception on the impact of rearing a child with ASD on their family life, and how these families function within the Bruneian sociocultural context.	A qualitative, in-person, semi-structured interview was conducted; with a quantitative self-report on the parents' perception of the impact of having a child with ASD on family function, as measured via Impact on Family Scale (IFS; Stein and Reissman, 2004).	Descriptive statistics yielded from the IFS, found the diagnosis of a child with ASD to highly affect the families' financial situation, social interaction within and outside the home, and the subjective distress experienced by the parents.<br><br>Results indicate that, whilst half of sampled families might be coping well with the added stress of having a child with ASD, the other half of the sampled family are on the contrary struggling to cope.<br><br>Sources of stress were suggested to include, a lack of financial support from the government, lack of awareness on ASD in society,

Study	Location	Design	Sample	Aim/Goal	Outcome Measures	Findings
						worries about their child's future, and inadequate/ limited access to educational and support services.<br><br>Content analyses conducted on the narrative data from the interviews found that the parents' Islamic faith played an important role as a coping strategy in raising a child with ASD.
Ting and Chuah (2010)	Malaysia	Qualitative study.	12 parents (8 mothers, 4 fathers) with children, who were registered at the Sarawak Autistic Association, Kuching.	To explore the experience and coping strategies among parents in raising a child with ASD in Kuching, Malaysia.	Nil.	Parents were initially in denial about their child's diagnosis, but carried on with usual daily routines (i.e., taking bath, using the toilet) when they no longer perceived it as a crisis. At this acceptance stage, the relationships between family members with the child with ASD were positive.<br><br>Parents trained their children using verbal instructions and demonstrations, though it remained a challenged to raise and nurture them.<br><br>Parents generally relied on religious beliefs and family support as coping strategies.

Study	Location	Design	Sample	Aim/Goal	Outcome Measures	Findings
						Generally, parents received negative views from the public in regard to having a child with ASD. Parents had problems in trying to place their child in pre-school or day-care. Findings also suggested the need for more support services in Sarawak for children with autism and for their parents.
Vetrayan, Daud and Paulraj (2013)	Malaysia	Quantitative study.	33 parents of children with autistic disorder, who were recruited from four different places: Hospital Tengku Ampuan Rahimah, Hospital Kuala Lumpur, National Autism Society of Malaysia, and Research Laboratory for Autism.	To determine the level of hopelessness among parents of children with autistic disorder, and if this is affected by the age of the child, and/or the parents' level of education.	Beck Hopelessness Scale (BHS; Beck and Steer, 1988).	Parents of children with autism experienced a mild level of hopelessness.<br><br>Feelings of hopelessness experienced by the parents were negatively correlated with parents' level of education and family income.<br><br>There was also a statistical difference among parents' level of hopelessness in respect to the type of day-care the child with autism was sent to (i.e., no day-care, half day-care, full day-care). However, no post-hoc analyses were run to determine where these differences lied.

Study	Location	Design	Sample	Aim/Goal	Outcome Measures	Findings
						Age of the child with ASD was not correlated to parents' level of hopelessness.
Wahyuni (2013)	Indonesia	A qualitative research was conducted with two single-mothers as case studies for one and a half months.	Two single mothers of children with autistic disorder.	To understand the mechanisms of self-regulation as a coping strategy in single mothers of children with autism.	For each case study, a guided interview, observation and field report methods were used.	Self regulation was found to be processed and utilised differently between the mothers; and this was found to be dependent on three primary aspects: the effort exhibited in attaining life's goal, the evaluation of life's goal and belief in self-efficacy.<br><br>Other supporting factors, which were found to contribute to self-regulation included: background differences in the occurrence and diagnosis of the child's diagnosis, their acceptance and adaptability towards their child's diagnosis with autistic disorder and finally, their interpretation and thus, understanding in response to having child with autistic disorder.<br><br>Other influencing factors were also noted: mother's age, number of children, mother's educational background, current job, income,

Study	Location	Design	Sample	Aim/Goal	Outcome Measures	Findings
						religious strength, family support, and environmental influences.
Wisessathorn, Chanuantong, and Fisher (2013)	Thailand	Quantitative study.	333 caregivers (237 mothers, 31 fathers; 48 grandparents; 17 others) of a child with ASD from autism centres and schools in Bangkok, Thailand.	To determine the relationship between severity of ASD symptoms on parental quality of life, and if this relationship is mediated by optimism.	The Childhood Autism Rating Scale (CARS; Schopler et al., 1980) was used to measure the severity of ASD in the child.<br><br>The WHOQOL-BREF (Group, 1998) test was used to measure parental Quality of Life, which includes four subscales: physical health, psychological health, social relationships and environmental factor.<br><br>The Life Oriented Test-Revised (LOT-R; Scheier, Carver, and Bridges, 1994) was used to measure optimism among parents.	A significant negative association was found between the severity of the child's ASD with parental quality of life.<br><br>Meanwhile, optimism was significantly positively correlated with parental quality of life in all domains.<br><br>Path analysis confirmed that optimism partially mediated this relationship, whereby, impairment of language and repetitive behaviour were found to be associated with optimism, which in turn was associated with parental quality of life.
Xue, Ooh, and Magiati (2014)	Singapore	Quantitative study.	65 Singaporean parents (46	To investigate the capabilities	Parental stress was measured using the	Parents reported relatively low levels of stress on the QRS-F scale.

Study	Location	Design	Sample	Aim/Goal	Outcome Measures	Findings
			mothers, 19 fathers), who were recruited from special schools, intervention centres, public hospitals, community centres, voluntary organisations, and private organisations.	(coping strategies and resources of social support) and positive meanings amongst parents of children with ASD, in an Asian context.	Parent and Family Problems and the Pessimism sub-scales of The Questionnaire on Resources and Stress – Friedrich Short Form (QRS-F; Friedrich et al. 1983). Parental stress was measured as a component of 'Total demands' exerted on the family as a result of having a child diagnosed with ASD.<br><br>Coping strategies was measured using The Coping Health Inventory for Parents (CHIP; McCubbin et al. 1981).<br><br>Positive meanings were measured via the Positive Family Impact (PFI) sub-scale of the Family Impact of Childhood Disability Scale (FICD; Trute,	Whilst majority of the parents reported aggressive and self-injurious behaviour to occur occasionally in their child, it was reported to cause little or only some interference with their lives.<br><br>Coping strategies that were found to be most helpful were family integration/ optimism; followed by understanding of the autism condition, and by acquiring esteem and psychological stability.<br><br>On average, parents reported two to three sources of social support; whereby, it mostly encompassed of support from immediate family; followed by school, other families of children with ASD, professionals and extended family.<br><br>Results also found total capabilities to positively correlate (with medium effect size) to family functioning. However, no significant relationship emerged between positive meaning and family functioning, though this was attributed to probable lack of power in the study.

Study	Location	Design	Sample	Aim/Goal	Outcome Measures	Findings
					Hiebert-Murphy, and Levine, 2007).<br><br>Family functioning was measured via four (i.e., Balanced Cohesion, Balanced Flexibility, Family Communication, and Family Satisfaction) of the eight subscales in The Family Adaptability and Cohesion Evaluation Scales IV (FACES IV; Olson, Gorall, and Tiesel, 2007).	Accordingly, reported total capabilities, (but not positive meanings) were found to mediate the relationship between demands and family functioning.
Yeo and Lu (2012)	Malaysia and China.	Quantitative study.	128 mothers (64 from Johor Bahru, Malaysia; and 64 from Hangzhou, China) of children with autistic disorder.	To compare parenting stress and psychological distress amongst mothers of preschool and elementary children with autistic disorder in Johor Bahru and Hangzhou.	The Childhood Autism Rating Scale Modified for Parents (CARS-P; Bebko, Konstantareas, and Springer, 1987) was used to measure mothers' perception of autism severity.<br><br>Parental Stress Scale (PSS; Berry and Jones, 1995) was used to	Mothers in Hangzhou were found to experience significantly higher levels of parenting stress and psychological distress in comparison to mothers in Johor Bahru. However, this was only true for mothers of children with ASD in preschool, and not in elementary school.<br><br>Specifically within Johor Bahru, there were no significant differences in parenting stress and

Study	Location	Design	Sample	Aim/Goal	Outcome Measures	Findings
				The study also aimed to determine the factors that were associated with parenting stress and psychological distress.<br><br>To determine the relationship between parenting stress and psychological distress among mother of children with autistic disorder in preschool and elementary school, in Johor Bahru as well as Hangzhou.	measure parenting stress.<br><br>The Depression Anxiety Stress Scale-21 (DASS-21; Lovibond and Lovibond, 1995) was utilised to measure psychological distress.	psychological distress experienced by mothers of children with ASD in preschool compared to those in elementary school.<br><br>Within Hangzhou however, mothers of children with ASD in preschool, experienced a significantly higher level of stress than mothers of children with ASD in elementary school.<br><br>Parenting stress among mothers in Hangzhou were found to be significantly influenced by perceived autism symptoms (to a greater extent), spousal relationship, father's income and treatment cost, whereas parenting stress among mothers in Johor Bahru were found to be significantly affected by perceived autism symptoms, spousal relationship and years since the child's diagnosis.<br><br>In terms of psychological distress, for mothers in Hangzhou, perceptions towards symptoms of autism, spousal relationship and father's income (to a lesser extent)

Study	Location	Design	Sample	Aim/Goal	Outcome Measures	Findings
						were found to be significant predictors. Whereas for mothers in Johor Bahru, spousal relationship and treatment cost were found to significantly predict psychological distress.<br><br>Therefore, whilst both mothers' perception of autism symptoms and spousal relationships were cross-cultural factors that affected parenting stress, the only cross-cultural factor found to affect psychological distress was spousal relationship.<br><br>A significant positive correlation was also found between parenting stress and psychological distress. Specifically, in both Johor Bahru and Hangzhou, higher parenting stress among mothers of children with ASD in *preschool*, was associated with higher psychological distress (i.e., stress, depression and anxiety).<br><br>However, when analysed in respect to mothers of children with autistic disorder in *elementary* school, higher parenting stress was

Study	Location	Design	Sample	Aim/Goal	Outcome Measures	Findings
						associated with higher psychological distress (i.e., stress and depression) only for mothers in Hangzhou, whereas in Johor Bahru, higher parenting stress was associated with higher depression (only).
