# Supplementary material for: Parenting Stress and Resilience in Parents of Children With Autism Spectrum Disorder (ASD) in Southeast Asia: A Systematic Review
Source: Front Psychol. 2018 Apr 9;9:280. doi: 10.3389/fpsyg.2018.00280 (PMC5900388; doi:10.3389/fpsyg.2018.00280)
Supplement: Supplementary file 2 [file Table2.pdf]

Supplementary Table 2. Characteristics of Parents Sampled in this Systematic Review

**Study**	***N***	**Age of Mother**	**Age of Father**	**Mothers' Education/ Employment Status**	**Fathers' Education/ Employment Status.**	**No. of Children**
Athari, Ghaedi and Kosnin (2013)	250 mothers.	Not stated.	n/a	Not stated.	n/a	Not stated.
Callos (2012)	20 parents (17 mothers, 3 fathers).	Including both mothers and fathers: age range = 20 – 35 years old (n = 12), more than 35 years old (n = 8).		Including both mothers and fathers: high school graduate (n = 6), college graduate (n = 14).<br><br>Including both mothers and fathers: working (n = 12), not working (n = 8).		Including both mothers and fathers: one to two children (n = 11), more than two children (n = 9).
Charnsil and Bathia (2010)	27 caregivers (24 parents, 3 relatives).<br><br>(15 females, 12 males).	Including all caregivers, *M* = 46.70 years, *SD* = 8.50 years.		Including all caregivers, years of education (*M* = 11.34, *SD* = 5.03		Not stated.

Study	*N*	Age of Mother	Age of Father	Mothers' Education/ Employment Status	Fathers' Education/ Employment Status.	No. of Children
Chong and Kua (2016)	10 mothers.	Age range = 34 – 51 years; *M* = 40.2 years,	n/a	Not stated. However, mother's occupation was reported: housewives (n = 4); middle–high income jobs (n = 6).	n/a	Including the child with ASD, one child (n = 5); two children (n = 5).
Foo, Yap and Sung (2014)	6 (3 mothers, 3 fathers).	Parents' age range = 36 – 49 years; *M* = 44 years.		Secondary education, n = 1; diploma, n = 2; bachelor's degree, n = 1; master's degree, n = 2<br><br>(All mothers in the study were housewives).		Not stated.
Foronda (2000)	16 single mothers: spouses were abroad for a long period of time due to work/study (n = 9); widows (n = 3); separated	Majority were 31 – 40 years (n = 13; 81.2%).	n/a	Majority had attended college (n = 11; 68.7%), with four of them (25%) having attended vocational school and one of them (6.3%) having studied to a master's level.	n/a	Including the child with ASD, majority (n = 7; 43.8%) had two children, followed by one child (n = 5; 31.3%), whereas an equal number of single mothers had three

Study	*N*	Age of Mother	Age of Father	Mothers' Education/ Employment Status	Fathers' Education/ Employment Status.	No. of Children
	(n = 3); unmarried (n = 1).			In terms of employment: housewives (n = 6; 37.5%); housewives but conducted part-time sales (n = 3; 18.8%); employed (n = 5; 31.3%); self-employed (n = 2; 12.5%).		children (n = 2; 12.5%), and four or more children (n = 2; 12.5%).
Ilias, Liaw, Cornish, Park and Golden (2016)	8 mothers	Age range = 32 – 59 years; *M* = 43.5 years.	n/a	High school (n = 2), pre-university (n = 1), diploma (n = 1), bachelor's degree (n = 2), master's degree (n = 2).<br><br>Employment status: housewife (n = 3), student (n = 1), full-time office work (n = 1), part-time work (n = 1), and part-time freelancer (n = 1).	n/a	Six mothers had one child with ASD, and two mothers had two children with ASD.<br><br>Number of other children was not reported, but the number of household members was reported: four (n = 4), five (n = 3), six (n = 1).

**Study**	***N***	**Age of Mother**	**Age of Father**	**Mothers' Education/ Employment Status**	**Fathers' Education/ Employment Status.**	**No. of Children**
Ha, Whittaker, Whittaker, and Rodger (2014)	27 parents (21 mothers, 6 fathers), among which, there were three couples.	Including both mothers and fathers, age range = 28 – 56 years old.		Including both mothers and fathers, majority had at least graduated with an undergraduate degree. Only three participants had high school level education.		All parents had only one child with ASD. Number of other children was not reported.
Lai, Goh, Oei, and Sung (2015)	136 parents. (110 mothers, 26 fathers).<br><br>73 parents (54%) had a child with ASD, whereas 63 parents (46%) had children with typical development.	Including both mothers and fathers, *M* = 43.68 years, *SD* = 6.36 years.		Including both mothers and fathers: university/ postgraduate (n = 50; 36.8%), polytechnic/ pre-university (n = 30; 22%), secondary/ vocational (n = 45; 33.1%), primary or below (n = 3, 2.2%), others (n = 8; 5.9%).		Of the total 136 parents, 5 (3.7%) parents reported having an additional child with ASD
Liwag (1989)	13 families (13 mothers, 12 fathers).	*M* = 35.8 years	*M* = 36.48 years	Families (mothers and fathers) were reported to be well educated, financially stable and		Average number of children in the family is three,

Study	*N*	Age of Mother	Age of Father	Mothers' Education/ Employment Status	Fathers' Education/ Employment Status.	No. of Children
				hold middle level to high level positions in their workforce.		including the child with autism.
Moh and Magiati (2012)	102 (85 mothers, 17 fathers).	Including both mothers and fathers, age range = 32 – 63 years (*M* = 39.8 years, *SD* = 5.02 years).		Vocational qualification (n = 5), Below tertiary education (n = 48), Tertiary education or above (n = 48).<br><br>One parent failed to provide a response on education qualification.		Out of the 99 responses received on birth order, instances in which the child with ASD is the: only child (n = 18), first child (n = 34), 2nd/3rd/4th child (n = 47).
Nikmat, Ahmad, Oon, and Razali (2008)	52 parents (34 mothers, 18 fathers).	24 parents (46.2%) were in age range = 21 – 30 years, whereas 28 parents were between 31 – 40 years of age.		34 parents (65.4%) completed tertiary education, while 18 (34.6%) of them completed up to their secondary education.<br><br>Majority of the parents (n = 31; 59.6%) worked in private sectors, followed by government sectors (n =		Not stated.

Study	*N*	Age of Mother	Age of Father	Mothers' Education/ Employment Status	Fathers' Education/ Employment Status.	No. of Children
				14; 26.9%) whereas some were housewives (n = 7; 13.5%).		
Quilendrino, Castor, Mendoza, Vea, and Castillo-Carandang (2015)	Phase 1 (Focus group discussion): 15 parents of 10 children with autism (9 mothers, 6 fathers).<br><br>Phase 2 (Survey): 41 parents (34 mothers, 7 fathers).	Including both mothers and fathers, Phase 1: *M* = 39 years, *SD* = 7 years.<br><br>Phase 2: *M* = 36 years, *SD* = 6 years.		Not stated. However, including both mothers and fathers, number of parents employed: Phase 1 (n = 7); Phase 2 (n = 23).		Number of children with autism: Phase 1: one (n = 8), two (n = 1); Phase 2: one (n = 41). Number of other children was not stated.
Rahman, Ismail, Jaafar, Fong, Sharip, and Midin (2012)	One couple (a father and a mother).	36 years.	39 years.	Did not complete secondary education.	Did not complete secondary education.	Two.
Rejani and Ting (2015)	20 parents (10 mothers, 10 fathers).	Including both mothers and fathers, *M* = 39.5 years.		Not stated.	Not stated.	Not stated.

Study	*N*	Age of Mother	Age of Father	Mothers' Education/ Employment Status	Fathers' Education/ Employment Status.	No. of Children
Resurreccion (2013)	10 couples (i.e., 10 mothers, 10 fathers).	Age range = 27 – 52 years (*M* = 43.6 years).	Age range = 28 – 54 years (*M* = 45.8).	Low SES = four families; Middle SES = three families; High SES = three families.		Not stated.
Roffeei, Abdullah and Basar (2015)	Memberships of the two Facebook groups: Autism Malaysia (n = 3972); Autism Children Club (n = 4, 094).<br><br>Including both groups, approximately 80% of members were parents, whilst remaining 20% were special education teachers, providers of ASD-related	Not stated.	Not stated.	Not stated.	Not stated.	Not stated.

Study	*N*	Age of Mother	Age of Father	Mothers' Education/ Employment Status	Fathers' Education/ Employment Status.	No. of Children
	products and services, health professionals, academic researchers, and other individuals who may be directly or indirectly related to children with ASD.					
Santoso, Ito, Ohshima, Hidaka, and Bontje (2015)	14 mothers.	M = 40.3 Years	Not included in study sample, though all mothers were married.	Homemaker (n = 9); Private employer (n = 2); Private business (n = 1); Dentist (n = 1); Lawyer (n = 1).	n/a	Not stated.

Study	*N*	Age of Mother	Age of Father	Mothers' Education/ Employment Status	Fathers' Education/ Employment Status.	No. of Children
Siah and Tan (2015)	96 parents (72 mothers, 24 fathers).	Including both mothers and fathers: 30 years or below (5.2%), 31 – 45 years (72%), 45 years or above (41.7%).		Including both mothers and fathers: none or primary school level (11.5%), secondary school level (49%), above secondary school level (39.6%).		Number of children with ASD: one (97.9%), two (2.1%).
Siah and Tan (2016)	92 parents (69 mothers, 23 fathers)	Including both mothers and fathers; below 45 years (58.3%), above 45 years (41.7%)		Including both mothers and fathers: secondary or below (49%), above secondary (39.6%).<br>Held a full or part-time job (41.9%).		Not stated.
Sian and Tan (2012)	47 parents (36 mothers, 11 fathers).	Including both mothers and fathers, 74% of the were aged 36 or above.		37% of the parents had completed an education beyond the secondary level.		Not stated.
Tait and Mundia (2012)	40 parents (30 mothers, 10 fathers) from 30 families.	Age range = 23 – 45 years.	Age range: 23 – 44 years.<br>Mean age during birth	8 mothers (26.6%) were homemakers, whereas majority of them (n = 21; 70%) held full-time working positions, and	As according to the Scale of Occupational Prestige (Jones, 1989), majority of fathers (n = 22) fell	Not stated.

Study	*N*	Age of Mother	Age of Father	Mothers' Education/ Employment Status	Fathers' Education/ Employment Status.	No. of Children
		Mean age during birth of child with ASD = 30.5 years.	of child with ASD = 32 years.	only one mother held an upper class working position (i.e., Director).	into categories of lower to upper-middle class.	
Ting and Chua (2010)	12 parents (8 mothers, 4 fathers)	Including both mothers and fathers, age range = 29 – 48 years.		Varied among parents; no further information were provided.		Not stated.
Vetrayan, Daud, and Paulraj (2013)	33 parents.	Including both mothers and fathers, age range = 31 – 60 years (*M* = 39 years, 5 months).		Including both mothers and fathers: Tertiary education (63.6%), secondary education (36.4%)		
Wahyuni (2013)	2 mothers.	Not stated.	n/a	Not stated.	n/a	Not stated.
Wisessathorn, Chanuantong, and Fisher (2013)	333 caregivers (237 mothers, 31 fathers; 48 grandparents; 17 others).	Including all caregivers, age range = 19 – 70 years (*M* = 43.17 years, *SD* = 9.73 years).		Including all caregivers: Master's degree and above (n = 40; 11.9%), bachelor degree (n = 118; 35.6%), high school diploma (n = 75; 22.4%), primary school		One child (55.2%), two-children (38.9%), three-children (4.3%), four-children (1.3%), five-children (0.3%).

**Study**	***N***	**Age of Mother**	**Age of Father**	**Mothers' Education/ Employment Status**	**Fathers' Education/ Employment Status.**	**No. of Children**
				(n = 84; 25.4%), did not respond (n = 16; 4.7%).		
Xue, Ooh, and Magiati (2014)	65 (46 mothers, 19 fathers).	*M* = 37.9 years (n = 45; one mother did not report her age).	*M* = 42.2 years (n = 18; one father did not report his age).	Including both fathers and mothers: undergraduate degree and above (n = 34; 53.1%), diploma or professional qualification (n = 16; 25%), 'A' levels and below (n – 14; 21.9%).		15 parents (23.1%) had the child with ASD as their only child; 42 (64.6%) parents had one more child in addition to the child with ASD, whereas 8 (12.3%) parents had more than one child in addition to their child with ASD.
Yeo and Lu (2012)	128 mothers (64 from Johor Bahru, Malaysia; and 64 from Hangzhou, China).	Majority (n = 71; 55.5%) were in the age range: 21 – 30 years.	n/a	Not stated.	n/a	Not stated.
